# The proteasome deubiquitinase inhibitor VLX1570 shows selectivity for ubiquitin-specific protease-14 and induces apoptosis of multiple myeloma cells

**DOI:** 10.1038/srep26979

**Published:** 2016-06-06

**Authors:** Xin Wang, Magdalena Mazurkiewicz, Ellin-Kristina Hillert, Maria Hägg Olofsson, Stefan Pierrou, Per Hillertz, Joachim Gullbo, Karthik Selvaraju, Aneel Paulus, Sharoon Akhtar, Felicitas Bossler, Asher Chanan Khan, Stig Linder, Padraig D’Arcy

**Affiliations:** 1 Department of Medical Health Sciences (IMH), Linköping University, S-751 85 Linköping, Sweden; 2 Cancer Center Karolinska, Department of Oncology and Pathology, Karolinska Institute, S-171 76 Stockholm, Sweden; 3 ESP Life Sciences Consulting AB, Box 119, 431 22 Mölndal, Sweden; 4 Biosynchro in West AB, Karl Johansgatan 142, 414 51 Göteborg, Sweden; 5 Department of Immunology, Genetics and Pathology, Uppsala University, 751 85 Uppsala, Sweden; 6 Department of Hematology and Oncology, Mayo Clinic, Jacksonville, FL, USA; 7 Department of Cancer Biology, Mayo Clinic, Jacksonville, FL, USA; 8 German Cancer Research Center, DKFZ, Heidelberg, Germany

## Abstract

Inhibition of deubiquitinase (DUB) activity is a promising strategy for cancer therapy. VLX1570 is an inhibitor of proteasome DUB activity currently in clinical trials for relapsed multiple myeloma. Here we show that VLX1570 binds to and inhibits the activity of ubiquitin-specific protease-14 (USP14) *in vitro,* with comparatively weaker inhibitory activity towards UCHL5 (ubiquitin-C-terminal hydrolase-5). Exposure of multiple myeloma cells to VLX1570 resulted in thermostabilization of USP14 at therapeutically relevant concentrations. Transient knockdown of USP14 or UCHL5 expression by electroporation of siRNA reduced the viability of multiple myeloma cells. Treatment of multiple myeloma cells with VLX1570 induced the accumulation of proteasome-bound high molecular weight polyubiquitin conjugates and an apoptotic response. Sensitivity to VLX1570 was moderately affected by altered drug uptake, but was unaffected by overexpression of BCL2-family proteins or inhibitors of caspase activity. Finally, treatment with VLX1570 was found to lead to extended survival in xenograft models of multiple myeloma. Our findings demonstrate promising antiproliferative activity of VLX1570 in multiple myeloma, primarily associated with inhibition of USP14 activity.

A diverse set of cellular processes such as cell cycle progression, DNA repair, metabolism and cell survival are dynamically controlled by the synthesis and degradation of protein regulators. In eukaryotic cells the regulated degradation of proteins is controlled mainly by the ubiquitin proteasome system (UPS)^1^.

The UPS is composed of a destruction tag in the form of the small protein ubiquitin and the 26S proteasome, a large multi-subunit proteolytic complex that specifically degrades ubiquitin tagged proteins into small peptides. The proteolytic activities of the proteasome reside within the 20S core particle (20S CP), a barrel like structure composed of 4 stacked heptameric rings (α_7_β_7_β_7_α_7_) associated with one or two 19S regulatory particles (19S RP)^2^,^3^. Protein degradation begins with the covalent tagging of substrates with multi-ubiquitin chains, an event that initiates traffic to the proteasome and subsequent capture by highly specific ubiquitin receptors located within the 19S RP. Once bound, substrates undergo a sequence of modifications including de-ubiquitination by proteasome associated deubiquitinases (DUBs), unwinding by the 19S RP ATPases and finally translocation into the 20S CP where they are degraded^4^. Several roles for proteasome DUBs have been proposed including a rescue mechanism for improperly or poorly ubiquitinated substrates, maintenance of ubiquitin homeostasis by ubiquitin recycling, and facilitation of protein degradation by removal of the sterically bulky ubiquitin chains^5^,^6^. The 19S RP contains three DUBs: two DUBs of the cysteine class (USP14 and UCHL5) located in the lid and one metalloprotease DUB (POH1) located at the base^5^,^6^,^7^. All three DUBs show some level of substrate preference with USP14 and UCHL5 showing activity towards the distal tips of ubiquitin chains and POH1 cleaving ubiquitin chain linkages *en bloc* from ubiquitinated substrates^5^.

Bortezomib (PS-341, Velcade®) and carfilzomib (Kypriolis®) are inhibitors of the 20S proteasome that are in clinical use for the treatment of patients with multiple myeloma and mantle cell lymphoma^8^,^9^,^10^. Genome-wide siRNA screens have indicated that proteasome inhibition promotes cell death by a number of mechanisms, including dysregulation of Myc, interference with protein translation and disruption of DNA damage repair pathways^11^. We recently showed that the small molecule b-AP15 interferes with the UPS by inhibiting the enzymatic activities of the proteasomal DUBs USP14 and UCHL5^12^. Dual inhibition of these DUBs is known to result in blocking of proteasome function^13^,^14^ and exposure to b-AP15 does indeed result in the accumulation of poly-ubiquitinated proteins in cells^12^. RA-9, a compound with a similar structure to b-AP15 (Supplementary Fig. 1), has also been demonstrated to inhibit proteasomal DUB activity and to inhibit tumor growth *in vivo*^15^. Other compounds have also been described to inhibit proteasome DUB activity, including auranofin^16^, WP1130^17^ and the curcumin analogue AC17^18^. A common feature of many of these compounds is the presence of α,β-unsaturated carbonyl groups that can potentially form covalent adducts with free thiols by Michael addition in the active site of cysteine DUBs^6^.

VLX1570 is an analogue of b-AP15 (Fig. 1a) that shows higher potency and improved solubility^19^. A phase 1/2 trial assessing the safety and efficacy of VLX1570 in patients with relapsed/refractory multiple myeloma is currently ongoing (NCT02372240). VLX1570 is a competitive inhibitor of proteasome DUB activity, with an IC_50_ of ~10 μM *in vitro*^19^. The *in vivo* IC_50_ for inhibition of proteasome DUB activity and induction of apoptosis is <1 μM, with multiple myeloma cells showing greater levels of sensitivity compared to other tumor types. The lower IC_50_ for activity *in vivo* is presumably due to rapid drug uptake and enrichment in cells^14^. However a number of issues related to the mechanism of action of VLX1570 remain, such as the demonstration of direct binding to proteasomal DUB enzymes, the effect of drug binding on proteasome structure, potential for drug resistance, a clearer understanding of the mechanisms of cell death and demonstration of *in vivo* antitumor activity. In this report we have addressed several of these issues. We show that USP14 is the preferential target of VLX1570 and suggest that the high expression of USP14 in multiple myeloma cells confers increased sensitivity to proteasome DUB inhibition with VLX1570.

## Results

### Preferential inhibition of USP14 by VLX1570

**Figure 1:**
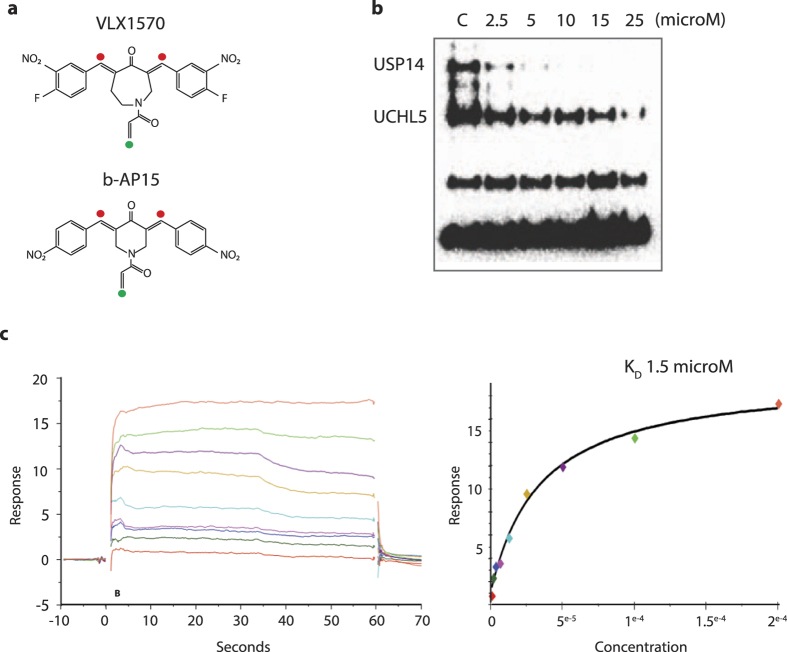
VLX1570 inhibits and binds to proteasome DUBs *in vitro*. (**a**) Structure of VLX1570 and b-AP15. The α,β-unsaturated carbonyls required for activity are marked with red filled circles and the Michael acceptor at the acrylamide is denoted with a green circle. (**b**) Inhibition of active-site-directed labeling of proteasomal deubiquitinases. Purified 19S proteasomes (5 nM) were pre-treated with DMSO or the indicated concentrations of VLX1570 (in DMSO) for 10 min at room temperature, followed by labeling with HA-Ub-VS and immunoblotting. (**c**) VLX1570 binding to USP14. Left: readings show binding signal in RU plotted against time in seconds. Higher concentrations clearly give rise to a higher binding signal. Right: points taken 10 seconds from the stop of injection in the reading to the left, plotted against the concentration on a linear scale. VLX1570 saturates the binding site in USP14 approaching a max signal and the fit of the data approaches the R_max_. The dissociation constant K_D_ is read from the fit where the 1:1 binding isotherm has the value of 50% of the R_max_.

VLX1570 is a bis-benzylidine azepane derived from the optimization of the hit molecule b-AP15 (Fig. 1a; Supplementary Fig. 1a)^12^,^19^. We have previously shown that VLX1570 preferentially inhibits proteasomal DUB activity while not inhibiting the activities of a panel of non-proteasomal DUBs^19^. Gene expression profiling showed that VLX1570 induces a stress response similar to b-AP15; however improved solubility characteristics and potency makes it more suitable for patient administration^19^. In order to investigate the DUB preference for VLX1570 we performed a dose-response experiment using increasing concentrations of VLX1570 on purified 19S RP labeled with an active site probe ubiquitin vinylsulphone (Ub-VS)^20^ (Fig. 1b). USP14 activity was strongly inhibited at 2.5 μM VLX1570 whereas UCHL5 inhibition required higher drug concentrations (Fig. 1b), suggesting that USP14 is particularly sensitive to VLX1570. VLX1570 contains two α,β-unsaturated carbonyls in a long conjugated system (Fig. 1a; red circles) and one acrylamide moeity (green circle) that can all function as potential Michael acceptors. Other DUB inhibitors described in the literature also have a similar α,β-unsaturated carbonyls chemical structure and are expected to react with cysteines in the active sites of DUB enzymes^6^,^15^,^17^,^18^,^21^,^22^ (Supplementary Fig. 1a). The acrylamide of b-AP15/VLX1570 has previously been shown to be redundant for anti-proliferative activity^14^,^19^, however the role of the specific α,β-unsaturated carbonyls are unknown. To address this question, an analogue (VLX1680, Supplementary Fig. 1b) was synthesized containing a single α,β-unsaturated carbonyl unit. VLX1680 was found to have lower antiproliferative activity and weaker DUB inhibitory activity compared to VLX1570 (Supplementary Fig. 1c,d), suggesting that both carbonyl units are required for optimal activity.

### Binding of VLX1570 to proteasome deubiquitinases *in vitro*

We next examined binding of VLX1570 to recombinant USP14 and UCHL5 using a surface plasmon resonance (SPR) approach. Recombinant proteins were immobilised on chip surfaces using Ni^2+^-histidine interactions. The dissociation constant (K_D_) for binding of VLX1570 to recombinant USP14 was between 1.5 and 18 μM using two different sources of recombinant protein (Table 1; Fig. 1c,d). The K_D_ for recombinant UCHL5 was higher compared to that of USP14 (14–18 μM), consistent with Ub-VS labeling showing preferential inhibition of USP14 compared to UCHL5 (Fig. 1b). Binding was reversible, consistent with previous studies showing reversible enzyme inhibition by b-AP15^12^,^14^. No saturable binding was observed using USP5 (data not shown). We next examined binding of VLX1570 to 26S proteasomes and recombinant USP14 using CM7 chips (Table 1). Immobilisation is here based on covalent unspecific binding to the chip surface. We could demonstrate specific, saturable binding to both recombinant USP14 and 26S proteasomes by VLX1570, although at somewhat higher K_D_, 35 μM and 43 μM respectively (Table 1). Interestingly, 26S proteasomes that were pre-incubated for 30 min at 37 °C with 10 times molar excess of the irreversible DUB inhibitor Ub-VS displayed a drasticallly lowered the B_max_ of VLX1570 (Table 2), suggesting that VLX1570 binds into the active site(s) of proteasome DUBs.

### Thermal stabilization of USP14 and UCHL5 in cells exposed to VLX1570

**Figure 2:**
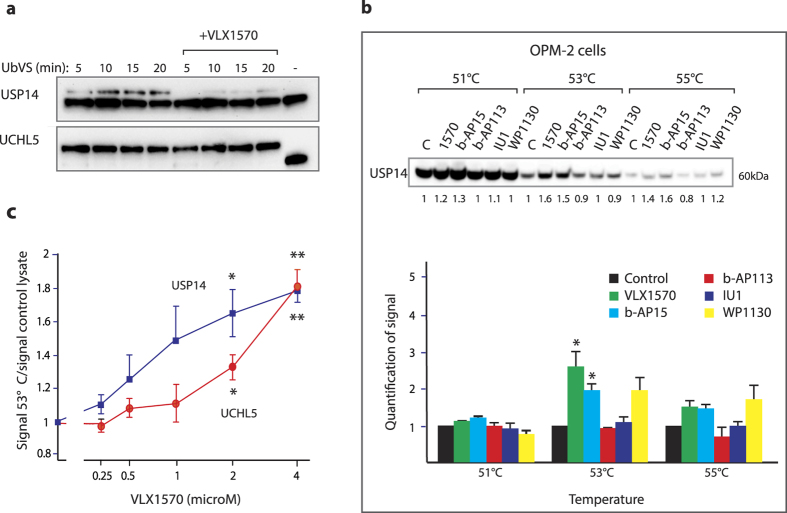
VLX1570 inhibits and binds to proteasome DUBs in exposed cells. (**a**) Inhibition of active-site-directed labeling of proteasomal deubiquitinases by VLX1570 in MM cells. OPM-2 MM cells were exposed to 0.5 μM VLX1570 for 3 hours and 25 μg whole cell lysates were subsequently labeled with Ub-VS (1 μM), followed by SDS gel electrophoresis and immunoblotting with USP14 or UCHL5 antibodies. The upper bands represent active USP14 or UCHL5 enzymes. (**b**) Thermo-stabilization of USP14 in exposed cells. The same number of OPM-2 MM cells were exposed to DMSO, 1 μM VLX1570, 1 μM b-AP15, 10 μM b-AP113, 20 μM IU1 or 5 μM WP1130 for 1 hour and analysed for thermostability of UPS14 by CETSA (cellular thermal shift assay)^23^. The lower panel shows means ± S.E.M. for 3 different experiments (*p < 0.05 by t-test; calculated relative to the inactive control b-AP113). (**c**) Dose-response of thermal stabilization of USP14. OPM-2 myeloma cells were exposed to different concentrations of VLX1570 for 1 hour and collected for CETSA analysis. Shown are means ± S.E.M. for 3 different experiments (*p < 0.05; **p < 0.01 by t-test).

We used Ub-VS to label active DUB enzymes in cells exposed to VLX1570 (Fig. 2a). The reversibility of VLX1570 binding is a complicating factor in this type of experiment, thus different Ub-VS labeling times were used. Inhibition of USP14 activity could be demonstrated in cells exposed to 0.5 μM VLX1570 whereas UCHL5 inhibition was weak in comparison (Fig. 2a). In order to provide evidence for binding of VLX1570 to proteasomal DUBs in living cells we used a cellular thermostabilization assay (CETSA)^23^. USP14 was thermostabilized after exposure of cells to 1 μM VLX1570 (Fig. 2b). Stabilization occurred at 53°–55 °C and was modest (2.5-fold control) but reproducible. The hit molecule b-AP15^12^ and the DUB inhibitor WP1130^17^ also induced thermostabilization of USP14 at 1 and 5 μM, respectively (Fig. 2b; structures shown in Supplementary Fig. 1a). In contrast, the inactive analogue b-AP113 did not induce thermostabilization of USP14. The effect of different drug concentrations on the thermostability of USP14 and UCHL5 was also investigated (using a single temperature in the CETSA) (Fig. 2c). Consistent with the findings of preferential inhibition of USP14 enzyme activity and consistent with the SPR data, USP14 was stabilized at lower concentrations of VLX1570 than UCHL5 in cells (Fig. 2c). We conclude from these experiments that VLX1570 preferentially binds USP14 both *in vitro* and in exposed cells.

### Knock-down of USP14 in multiple myeloma cells induces loss of cell viability

**Figure 3:**
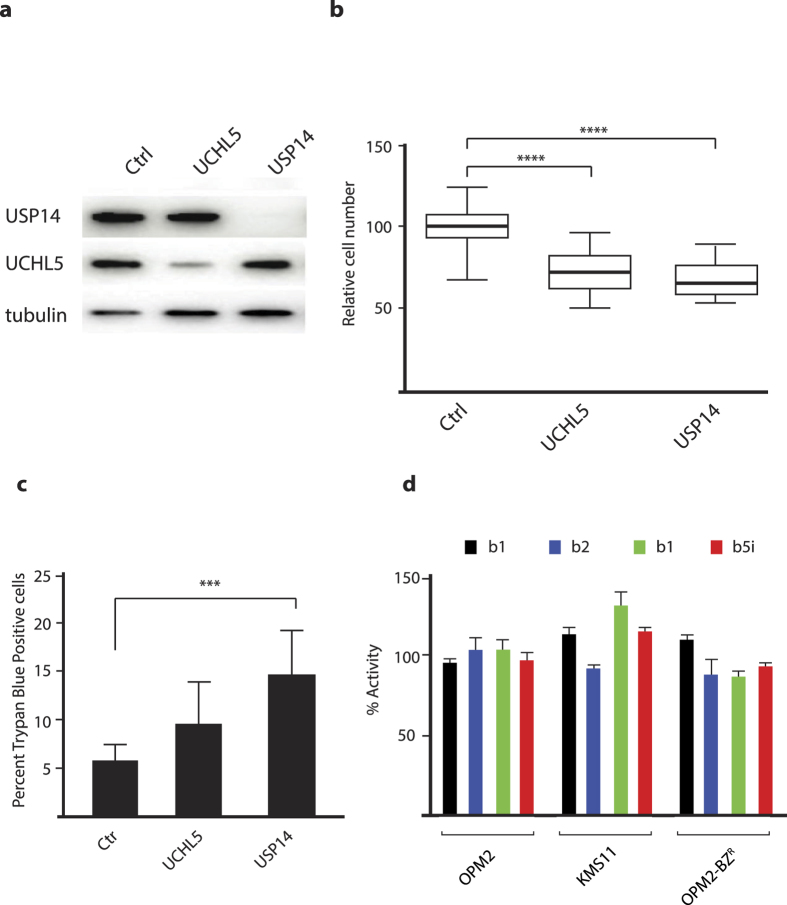
Knockdown of USP14 or UCHL5 results in loss of myeloma cell viability. OPM-2 cells were transfected with siRNAs to USP14, UCHL5 or scrambled siRNAs. (**a**) Protein expression in transfected cells was determined using Western blotting after 72 hours; (**b**) the number of cells in different transfected cultures were determined. Shown are box-plots (median, quartiles and 10th and 90th percentiles). Differences were significant at the level of p < 0.0001 (Wilcoxon). (**c**) the number of dead cells was determined in transfected cultures 72 hours after transfection using Trypan Blue staining (p < 0.0005; Student’s t-test); (**d**) VLX1570 does not inhibit proteasome or immunoproteasome activity. OPM-2 cells were exposed to 0.5 μM VLX1570 for 3 hours and extracts were assayed using different proteasome substrates.

We next used an siRNA approach to knock down the expression of USP14 and UCHL5 in multiple myeloma (MM) cells (Fig. 3a). Downregulation lead to reduction in cell number and a decrease in overall cell viability, presumably due to the essential role of these DUBs in maintaining proteostasis (Fig. 3b,c). Next we determined if siRNA depletion of either DUB altered the proteolytic activity of the proteasome. siRNA knockdown of either DUB did not affect the chymotryptic activity of the 20S CP (Supplementary Fig. 3). Finally, to rule out off-target effects of VLX1570, we analyzed the chymotryptic, tryptic and caspase-like activity of the 26S proteasome and the induced chymotryptic activity of the immunoproteasome in multiple myeloma cells. Consistent with our previous data no change in the proteolytic capacity of conventional or immunoproteasome was observed, supporting the notion that inhibition of proteasome DUB activity targets an upstream pathway (Fig. 3d).

### VLX1570 induces accumulation of polyubiquitin chains and elicits apoptosis of multiple myeloma cells

**Figure 4:**
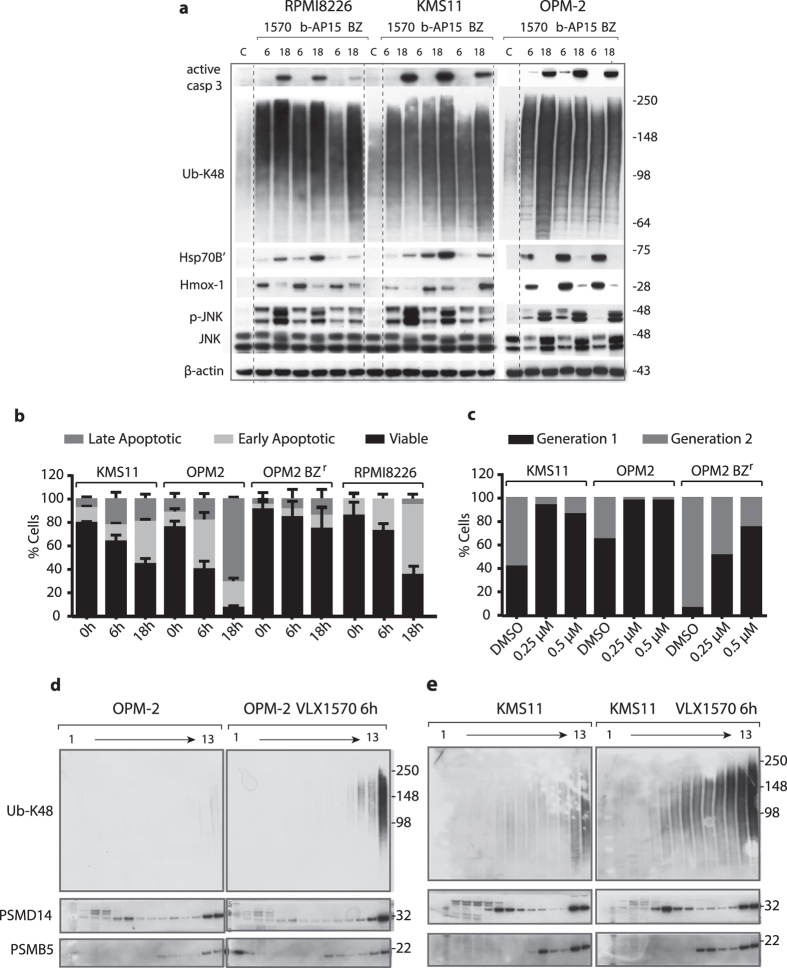
VLX1570 induces proteasome-associated polyubiquitin accumulation and apoptosis of multiple myeloma cells. (**a**) MM cells were exposed to 0.5 μM VLX1570, 0.5 μM b-AP15 or 50 nM bortezomib (BZ) for 6 or 18 h, followed by immunoblotting for active caspase-3, Ub-K48, HSP70B  ´(HSPA6), Hmox-1, phospho-JNK, JNK and β-actin. RPMI8826 and KMS11 data are from the same filter, OPM-2 from a separate (dashed lines were introduced for clarity). (**b**) Determination of cell death induction after exposure to 0.5 μM VLX1570 or vehicle for 0, 6 or 18 h. Cells were stained with FITC-conjugated annexin V and propidium iodide and processed by flow cytometry. The percentage of viable, early apoptotic and late apoptotic were quantified. Results shown are mean values of triplicate measurements. (**c**) Estimation of cell division based on dilution of carboxyfluorescein succinimidyl ester (CFSE) membrane staining during cell division. Cells were exposed to 0.25 μM or 0.5 μM VLX1570 or DMSO as a control for 72 h. The fraction of cells in different generations was determined by flow cytometry analysis. (**d,e**) Analysis of the effects of VLX1570 on the sedimentation profiles of polyubiquitinated proteins. Cell lysates from control or VLX1570-exposed OPM-2 (**c**) or KMS-11 cells (**d**) were subjected to glycerol gradient centrifugation. Gradient fractions were collected (1 = top; 13 = bottom) and subjected to immunoblotting using antibodies to Ub-K48, PSMD14 (19S subunit), or PSMB5 (20S subunit). Note the accumulation of polyubiquitin on 26S proteasomes after 6 hours of drug exposure.

Multiple myeloma (MM) cells are known to be sensitive to inhibition of proteasomal activity^24^ and we previously demonstrated that MM cells are sensitive to b-AP15^25^. Interestingly, examination of gene expression profiles of different cancer cell lines (Broad-Novartis Cancer Cell Line Data Base) showed that USP14 and UCHL5 are both strongly expressed in MM cells (Supplementary Fig. 4a). We examined the response of three human MM cell lines to VLX1570, b-AP15 and to bortezomib. All cell lines showed IC_50_ values in the submicromolar range in response to VLX1570/b-AP15 (Table 3). Apoptosis was observed in these cell lines as evidenced by activation of caspase-3, surface exposure of annexin V and loss of mitochondrial membrane potential (Fig. 4a,b; Supplementary Fig. 4b). We also examined the proliferative capacity of cells that remained viable after exposure to VLX1570. Cells were labeled with the fluorescent membrane dye CSFE, exposed to VLX1570 and the distribution of dye to daughter cells assessed by flow cytometry after 72 hours incubation. VLX1570 was found to arrest myeloma cells in the cell cycle (Fig. 4c).

Consistent with a block of proteasome function, polyubiquitinated proteins accumulated in VLX1570-treated MM cells (Fig. 4a). This was paralleled by increases in the inducible form of the chaperone Hsp70 (Hsp70B′) (Fig. 4a). We also observed induction of Hmox-1, an Nrf2 target gene and a marker of oxidative stress^26^, in VLX1570-exposed cells (Fig. 4a). We previously observed that b-AP15, similar to bortezomib, increases the phosphorylation of JNK in colon cancer cells^27^. A similar response was observed by b-AP15 and VLX1570 in MM cells (Fig. 4a). In addition we observed induction of XBP-1s (a splice variant of X-box binding protein-1 and a marker of ER stress^28^) in MM cells (Supplementary Fig. 4c) suggesting activation of an ERAD response. We conclude that VLX1570 induces a set of markers characteristic of proteotoxic stress, ER stress and oxidative stress in MM cells.

### Polyubiquitin chains are associated with proteasomes

The small molecule RA190, with a similar structure to b-AP15/VLX1570 (Supplementary Fig. 1a), was shown to induce accumulation of polyubiquitin in cells^29^. This drug was found to bind and block the ubiquitin receptor ADRM1 (Rpn13). This observation prompted us to examine whether b-AP15/VLX1570 blocks polyubiquitin association with proteasomes. K48-linked polyubiquitin chains were, however, found to cosediment with 26S proteasomes in extracts from KMS-11 and OPM-2 myeloma cells exposed to VLX1570 (Fig. 4d,e). PSMD14 (POH1, 19S RP) and PSMB5 (20S CP) subunits showed the same sedimentation patterns in control and drug exposed cells (Fig. 4d,e, Supplementary Fig. 4d), suggesting that gross proteasome structure was not affected by drug exposure. Whereas UCHL5 was present in high molecular weight complexes (presumably 19S and 26S), USP14 was not associated with 26S proteasomes in control cells or drug-exposed cells (Supplementary Fig. 4d). USP14 reversibly associates with the Rpn1 subunit of the 19S RP base^20^,^30^,^31^, and presumably dissociated from the proteasomes during centrifugation. To further examine whether proteasome structure was affected by VLX1570, we purified proteasomes from a HEK293 cell line expressing His-tagged Rpn11^31^. Both USP14 and UCHL5 were present in proteasome preparations from these cells and exposure to VLX1570 did not alter the yield of these proteins (Supplementary Fig. 4e). We conclude from these experiments that VLX1570 does not inhibit binding of polyubiquitin to proteasomes and does not induce gross alterations in proteasome structure.

### VLX1570 sensitivity is affected by drug uptake but not by deficiencies in apoptosis mechanisms

**Figure 5:**
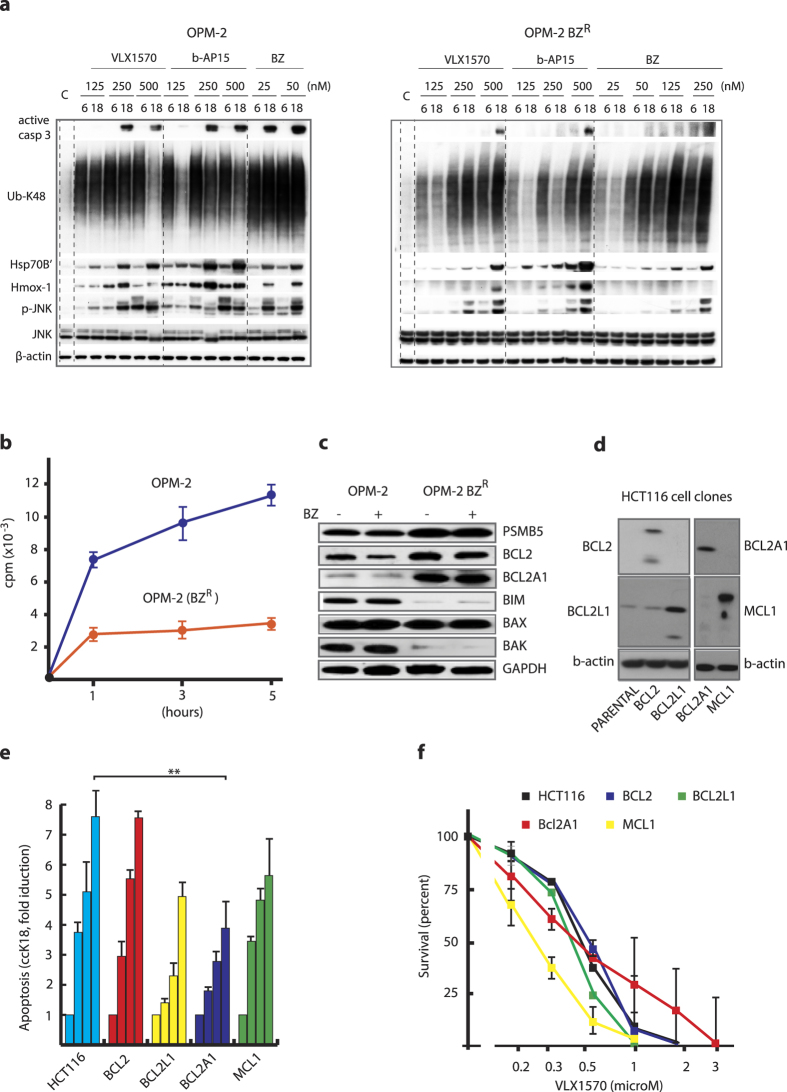
Examination of mechanisms affecting cellular sensitivity to VLX1570. (**a**) Characterization of the bortezomib-resistant myeloma cell line OPM-BZ^R^. Cells were exposed to VLX1570, b-AP15 or bortezomib (BZ) at the indicated concentrations for 6 or 18 h, followed by immunoblotting with Ub-K48, Hmox-1, HSP70B′, active-caspase-3, phospho-JNK, JNK, or β-actin antibodies. OPM-2 and OPM-2BZ^R^ samples are from separate filters, for comparision on the same filters see Supplementary Fig. 5d; (**b**) Uptake of ^3^H-VLX1570 by OPM-2 and OPM-2-BZ^R^ cells. Cells were incubated with labeled drug in growth medium at 37 °C, washed and processed for liquid scintillation counting. (**c**) Expression of apoptotic regulators and PSMB5 in OPM-2 and OPM-2 BZ^R^ cells. Note that long-term selection in the presence of bortezomib resulted in altered expression of BCL2, BCL2A1, BIM and BAK but that the expression of these proteins is not affected by acute drug exposure. PSMB5 is a 20S proteasome subunit, the expression of which is known to be associated with bortezomib resistance^52^. (**d**) Expression of BCL2-family proteins in HCT116 cell clones (full images are shown in Supplementary Fig. 8); (**e**) Apoptosis induction of HCT116 cells infected with lentiviruses expressing different BCL2 family members following exposure to 1 μM VLX1570 for 18 hours. Accumulation of caspase-cleaved K18 fragments in cells and culture media was measured by ELISA^51^. (**f**) Survival of HCT116 cells infected with lentiviruses expressing different BCL22 family members and exposed to different concentrations μM VLX1570. Survival was measured at 48 hours.

Exposure of cancer cells to cytotoxic drugs almost invariably leads to the development of drug resistance. We were interested in investigating mechanisms that would confer resistance to VLX1570. Continuous culture of myeloma cells in the presence of sublethal doses of VLX1570 did not result in cell populations with detectable resistance (Supplementary Fig. 5a,b). The observation that OPM-Bz^R^ (bortezomib resistant) cells show somewhat decreased sensitivity to VLX1570 and b-AP15 (Table 3) prompted us to investigate the mechanism involved. We did not observe decreased DUB inhibition in OPM-Bz^R^ cells by VLX1570 (Supplementary Fig. 5c), whereas lower levels of polyubiquitin accumulation and induction of stress markers were observed in these cells in response to VLX1570/b-AP15 (Fig. 5a; Supplementary Fig. 5d). We examined cellular uptake of VLX1570 using radiolabeled drug and found decreased uptake in the OPM-Bz^R^ cells compared to parental cells (Fig. 5b). We conclude that although only limited resistance to VLX1570 was obtained during prolonged culture in the presence of drug, decreased VLX1570 uptake can occur in some cells and affect the sensitivity to the drug.

OPM-Bz^R^ cells overexpress the apoptotic regulators BCL2 and BCL2A1, and express low levels of BIM and BAK (Fig. 5c). Apoptosis was nevertheless induced in these cell by VLX1570, albeit with a lower efficiency (Fig. 4b, Supplementary Fig. 5e). To further investigate the potential role of apoptotic regulators in VLX1570-induced apoptosis, we infected HCT116 cells with lentiviruses encoding BCL2 family members (Fig. 5d–f). Consistent with previous observations^12^, we found that BCL2 overexpression does not affect the induction of caspase-cleavage activity by b-AP15 in these cells and the same result was obtained using VLX1570 (Fig. 5e). Overexpression of BCL2A1 resulted in ~ 50% inhibition of caspase-cleavage activity (Fig. 5e). However, overexpression of BCL2A1 did not significantly affect cell survival over 72 hours following drug exposure (Fig. 5f). This observation suggested that cell death was apoptosis-independent and we therefore examined whether inhibition of total caspase activity using the pharmacological inhibitor z-VAD-fmk would affect survival of HCT116 cells exposed to VLX1570. We found that z-VAD-fmk only marginally increased cell viability (Supplementary Fig. 5f). These results suggest that defects in apoptotic pathways appear to be of minor importance for conferring resistance to VLX1570. We finally examined whether two inhibitors of necroptotic cell death^32^ increased the viability of cells exposed to VLX1570 and found this not to be the case (Supplementary Fig. 5f).

### Retention of VLX1570 in exposed cells

**Figure 6:**
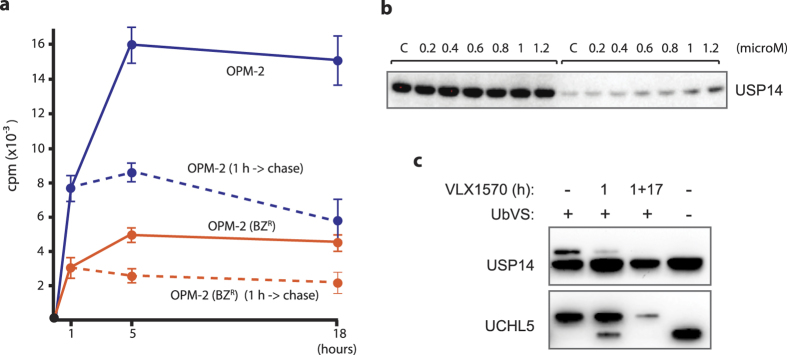
Retention of VLX1570 and target engagement after drug wash-out. (**a**) OPM-2 and OPM-2-BZ^R^ cells were exposed to VLX1570 for 1 hour and the uptake of ^3^H-VLX1570 was determined immediately or after an additional 17 hours incubation in drug-free medium; (**b**) Thermostabilization of USP14 was determined 17 hours after wash-out of VLX1570 by CETSA. Equal number of cells were used at each time point (loading controls are not relevant in this type of analysis^23^), (**c**) DUB inhibition was determined by Ub-VS labeling of cell extracts after 1 h exposure to VLX1570 and after 1 h exposure followed by 17 hours wash-out.

Cells become irreversibly committed to cell death when exposed to b-AP15, a phenomenon believed to be due to the retention of the drug in cells^14^. We examined the retention of VLX1570 in OPM-2 cells after 1 hour exposure and 17 hours incubation in drug-free medium. Approximately 70% of the drug was found to be associated with the cells after wash-out (Fig. 6a). VLX1570 was also retained in OPM-Bz^R^ cells (Fig. 6a). The observation of drug retention suggested that despite reversible binding of VLX1570 to its target, an intracellular pool remains over time which is sufficient for continuous target engagement. We indeed found that USP14 remained thermostabilized 17 h after wash-out of drug following a 1 hour exposure to VLX1570 and that USP14 was still inhibited at this time (Fig. 6b,c).

### *In vivo* studies of the effects of VLX1570 on multiple myeloma cells

**Figure 7:**
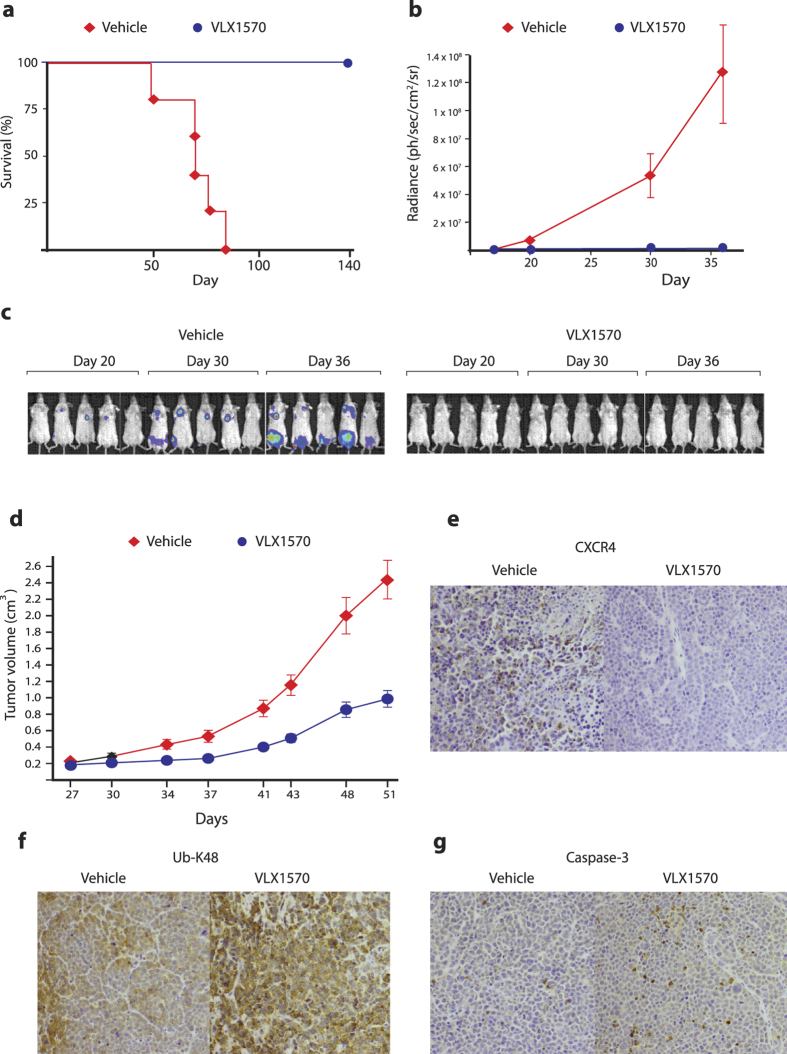
*In vivo* activity of VLX1570 in multiple myeloma xenografts. (**a**) KMS-11-LUC2 cells (5 × 10^6^) were injected intravenously into female SCID mice. After 7 days, mice were randomized into control and treatment groups (5 mice per group). Mice were treated with vehicle or VLX1570 (3 mg/kg) for 10 consecutive days. The drug was dissolved in PEG/Chremophore/Tween (50/10/40) and diluted 1:10 with saline prior to intravenous injection. Shown is survival over a 142 day period. (**b**) Quantification of bioluminescence measured at 18, 20, and 30 and 36 days of tumor cell injection. (**c**) Bioluminescence recorded in individual animals. (**d**) Growth of subcutaneous RPMI8226 tumors following treatment with VLX1570. Mice were exposed to VLX1570 dissolved in PEG/Chremophore/Tween (50/10/40). Mice were treated with 3 mg/kg VLX1570 for 10 consecutive days (5 mice per group). (**e**) Immunohistochemical staining showing decreased levels of CXCR4 in RPMI8226 tumors after exposure to 3 mg/kg VLX1570. (**f)** Increased immunohistochemical staining of K48-linked ubiquitin chains in RPMI8226 tumors after exposure to 3 mg/kg VLX1570. (**g**) Increased caspase-3 activity after exposure of RPMI8226 tumors to 3 mg/kg VLX1570.

Antineoplastic activity was examined using KMS-11 multiple myeloma cells growing orthotopically in immunocompromised mice. Seven days after intravenous injection of cells, mice were randomized into treatment groups. Treatment was then initiated and continued for 10 days using 3 mg/kg VLX1570. As shown in Fig. 7a, VLX1570 treatment increased the survival of the animals. The recorded luminescence is shown in Fig. 7b and individual animals in Fig. 7c. Importantly the anti-tumor activity of VLX1570 was not associated with reduction in body weight (Supplementary Fig. 7a). A similar treatment schedule was used to treat the subcutaneous RPMI8226 multiple myeloma model. As shown in Fig. 7d, VLX1570 caused a significant decrease in tumor growth in this model. The levels of the chemokine receptor CXCR4 are known to be regulated by USP14^33^ and we examined CXCR4 levels in RPMI8226 tumors by immunohistochemistry. We indeed found decreased CXCR4 staining after exposure to VLX1570 (Fig. 7e). We also observed increases in K48-linked ubiquitin chains and increased active caspase-3 after VLX1570 treatment of mice with RPMI8226 tumors (Fig. 7f,g). Finally, we observed decreased ERK phosphorylation in RPMI8226 cells *in vivo* after exposure of mice to VLX1570 (Supplementary Fig. 7b). Inhibition of ERK phosphorylation was observed in cells exposed to VLX1570 *in vitro* and was paralleled by decreased activation of RAS (Supplementary Fig. 7b).

## Discussion

VLX1570 belongs to a recently described class of drugs that inhibit proteasome deubiquitinase activity, resulting in impaired proteasome processing^12^,^15^,^16^,^18^,^19^. VLX1570 has a more favorable solubility profile and is more potent compared to the hit compound b-AP15. The drug has been approved for clinical studies by the US FDA and a phase 1/2 trial assessing the safety and efficacy of VLX1570 in combination with dexamethasone in patients with relapsed/refractory MM is currently ongoing (NCT02372240). We here show that, similar to b-AP15^25^, VLX1570 induces apoptosis and cell death of MM cells *in vitro* and has anti-neoplastic activity *in vivo*^25^. We show that VLX1570 induces the expression of the chaperone HSP70B′, the oxidative stress marker Hmox-1, and the ER stress marker XBP-1s. We also show downregulation of ERK phosphorylation in cultured cells and in MM tumors *in vivo*. The decrease in ERK phosphorylation *in vivo* was robust and good quality antibodies are available from different sources.

Both the SPR binding and the enzyme inhibition results showed stronger inhibition of USP14 compared to UCHL5. Furthermore, whereas the dose-response for thermal stabilization of USP14 was similar to that observed for proteasome inhibition and apoptosis induction, little or no stabilization was observed for UCHL5 at submicromolar doses. USP14 is strongly expressed in MM cells (Supplementary Fig. 4a) and has also been reported to be overexpressed in other malignancies such as ovarian and hepatocellular carcinoma^34^,^35^. We found that knock-down of either USP14 or UCHL5 in multiple myeloma cells resulted in loss of cell viability, consistent with our previous results^36^ and those of other investigators using hepatocellular carcinoma and ovarian cancer cells^34^,^35^. These findings raise the possibility that the antiproliferative activity of VLX1570 towards MM cells is due to a combination USP14 overexpression and the general susceptibility of MM cells to proteasome inhibition^37^,^38^. The role of USP14 appears, however, to be cell type-specific and complex. For example, the small molecule USP14 inhibitor IU1 stimulates proteasome degradation in mouse embryo fibroblasts^39^ and accelerates the degradation of cellular prion proteins^40^. USP14 is important for the function of neuronal cells since USP14 mutant mice (*ax*^J^/*ax*^J)^ mice) develop severe tremors and die by 6–10 weeks of age^41^ and mice expressing catalytically defective USP14 in the nervous system showed increased accumulation of polyubiquitin in the targeted cells^42^. These different observations could be explained by different cell-type specific requirement for USP14.

A number of inhibitors containing thiol-reactive α,β-unsaturated carbonyl groups have been described^12^,^15^,^17^,^18^,^19^,^21^,^43^ which are expected to target active site cysteines in DUBs. We indeed found that pretreatment of proteasomes with the irreversible DUB inhibitor Ub-VS decreases binding of VLX1570 to proteasomes. Small molecule DUB inhibitors generally show limited specificity for individual enzymes^44^ and thiol-reactive compounds are expected to be particularly promiscuous. VLX1570 does, however, not show inhibitory activity on a panel of different DUB enzymes^19^ or of total cellular DUB activity (our unpublished data) and the related b-AP15 and RA-9 compounds do also not inhibit total cellular DUB activity^12^,^15^. Our SPR experiments demonstrated reversible binding, consistent with previous findings of reversible enzyme inhibition^14^. Contrary to conventional wisdom, the reactions of a,β-unsaturated ketones and aldehydes with glutathione are known to be reversible^45^,^46^. We previously reported that b-AP15 is only slowly lost from cells after drug wash-out and suggested this phenomenon to explain the irreversibility of drug effects on cells^14^. We here found that VLX1570 is also retained in cells after removal of drug and that USP14 was engaged by drug 17 hours after wash-out, as evidenced by thermal stabilization and persistent enzyme inhibition. These findings provide an explanation for how a reversible enzyme inhibitor can generate irreversible inhibition in exposed cells.

We previously reported that b-AP15 is able to overcome bortezomib-resistance^25^. It was important to evaluate the extent of expected resistance towards VLX1570 and which mechanisms that would be involved. Interestingly, we have not been successful in developing cells that are highly resistant to VLX1570. A colon cancer cell line with a limited degree of resistance has been isolated in which resistance could be reversed by glutathione depletion (our unpublished results). The α,β-unsaturated ketones are expected to react with glutathione^45^,^46^ and this finding was therefore expected. The difficulty to derive resistant cells by direct selection prompted us explore the serendipitous finding of a bortezomib-resistant MM cell line which shows marginal cross resistance to VLX1570/b-AP15. These cells were produced by prolonged exposure to increasing concentrations of bortezomib and their clinical relevance may be limited. Our results did show, however, that VLX1570 uptake was diminished in this cell line. Gene expression profiling did not give any guidance to possible mechanisms of altered drug transport in the OPM-2BZ^R^ cells and we did not find competition between bortezomib and VLX1570 in drug uptake experiments (our unpublished data). Dysregulated apoptosis signaling would potentially be a mechanism of VLX1570 resistance and the OPM-2BZ^R^ cells examined here overexpress BCL2 and BCL2A1, and also express low levels of BIM and BAK. VLX1570 and b-AP15 were capable of inducing apoptosis of these cells, albeit at a lower efficiency. We previously reported that BCL2 overexpression does not lead to b-AP15-resistance in colon cancer cells^12^ and we here extended these studies by overexpressing different BCL2 family proteins. We found decreased apoptosis of cells overexpressing BCL2A1, a protein also overexpressed in OPM-2-BZ^R^ cells. BCL2A1 overexpression did not, however, affect cell survival. Since treatment with a pan-caspase inhibitor also had only limited effects on survival of VLX1570-exposed cells, we examined whether inhibitors of necroptosis (necrostatins) have any effects and found this not to be the case. We conclude that glutathione-mediated detoxification and drug uptake mechanisms are able to confer limited resistance to VLX1570, whereas defects in apoptosis signaling appears to be of minor importance. The observations of limited development of resistance is intriguing and is encouraging with regard to potential future clinical use.

The chalcone-derivative RA190 was reported to inhibit proteasome function by irreversible binding to the ubiquitin receptor ADRM1/Rpn13^29^. RA190 has a similar structure as b-AP15 and VLX1570 (Supplementary Fig. 1a). In addition to binding ubiquitin, ADRM1/Rpn13 also serves as a receptor for UCHL5^47^. We examined the possibility that exposure to VLX1570 leads to impaired binding of K48-linked polyubiquitin chains to the proteasome and found this not to be the case. Overall proteasome structure and UCHL5 association to proteasomes was also not affected. The discrepancies between our findings using VLX1570 and those using RA190 are interesting and suggest that structurally related chalcone-derivatives affect the UPS by different mechanisms.

Tumor cells are dependent on a functional ubiquitin-proteasome system, making it an attractive target for the development of cancer therapeutics. Drugs that inhibit the DUB activity of the 19S proteasome can potentially be used as second line therapy for patients that do not respond to conventional proteasome inhibitors. The low level of resistance development and the extended intracellular retention times of VLX1570 are attractive features for potential future use of this agent as an anti-cancer drug.

## Experimental procedures

### Reagents

b-AP15, VLX1570 and other analogues were synthesized by OnTarget Chemistry AB (Uppsala, Sweden). ^3^H-VLX1570 was synthesized by OnTarget Chemistry and had a specific activity of 2.0 MBq/mmol (radiochemical purity >97%). 19S proteasome (E-366), Ubiquitin-AMC (U-550), Ubiquitin Vinyl Sulfone (U-202), HA-Ubiquitin Vinyl Sulfone (U-212) (Boston Biochem, Cambridge, MA). Anti-β actin (AC-15), anti-HSPA6 (HPA028549) (Sigma Aldrich, St Louis, MO); anti-Ubiquitin K48 (Apu2) (Millipore); anti-Heme Oxygenase 1 (610712), anti PARP (556362), anti-active Caspase3 (559565); anti-USP14 (A300-919A) (Bethyl Laboratories); anti-HA (12CA5)(Roche); anti-Phospho-SAPK/JNK (4668), anti-Phospho-p38 Map Kinase (9211), anti-Phospho-p44/42 MAPK (Erk1/2) (4370)(Cell Signaling); anti-JNK (SC-571), anti-Erk1(K-23), anti-Erk2 (C-14), XBP-1s (ER stress sampler kit, Cell Signaling Technologies).

### Cell culture

HCT116 colon carcinoma cells were maintained in McCoy’s 5A modified medium/10% fetal calf serum. RPMI8226, KMS11, OPM-2 and OPM-2-BZ^R^ were maintained in RPMI1640 medium supplemented with 10% fetal calf serum. All cells were maintained at 37 °C in 5% CO_2_.

### Western blot analysis

Cell extract proteins were resolved by Tris-Acetate PAGE gels (Invitrogen, Carlsbad, CA) and transferred onto a polyvinylidene difluoride (PVDF) membrane for western blotting. For Ub-VS labeling, we lysed cell pellets from control or treated cells with buffer (50 mM HEPES pH 7.4, 250 mM sucrose, 10 mM MgCl2, 2 mM ATP, 1 mM DTT and 1%NP-40) on ice for 30 min and removed debris by centrifugation. We labeled 25 μg of protein with 1 μM Ub-VS for 30 min at 37 °C. We resolved samples by SDS-PAGE and performed immunoblotting. For HA-Ub-VS labeling, we pretreated purified 19S (5 nM) with DMSO or 2.5, 5, 10, 15 or 25μM 1570 for 10 min at room temperature, which followed with labeling with 1 μM HA-Ub-VS for 30 min at 37 °C and immunoblotting.

### CEllular Thermal Shift Assay (CETSA)

For CETSA, the same amount of cells as one sample was treated with required condition and collected as described^48^. Each sample was suspended in PBS supplemented with protease inhibitors after they were washed in PBS. And then these samples were heated at 51, 53, or 55 °C for 3 min. Immediately after heating, tubes were removed and incubated at room temperature for 3 min, and then snap-frozen in liquid nitrogen. Samples were freeze-thawed twice and centrifuged at 20,000 g for 20 min at +4 °C and supernatants were analysed by Western-blotting.

### Surface plasmon resonance

A BIACORE T200 was used for characterization of the interaction between compounds and USP14, UCHL5 and 26S proteasome. The standard BIACORE protocols were used for NTA chip (Ni/His-tag immobilization) and CM7 chip for compound interaction studies with slight modification described below. The x6 his-tagged USP14 and UCHL5 proteins were coupled to a Series S NTA BIACORE sensor chip in the BIACORE T200 using the his-tags for adherence of the proteins to the surface and an amine coupling procedure for covalent attachment to the chip surface. NTA chip was cleaned using three 500 μM EDTA injections followed by an injection of 500 μM Ni^2+^ and activation of the chip surface with EDC/NHS injection according to BIACORE standard methods. Each chip has four separate areas, which were handled separately during the immobilization procedure. Area 1 was used as blank reference surface, area 2–4 was coupled with investigated recombinant proteins or complexes. Proteins were diluted in running buffer (10 mM HEPES, 150 mM NaCl, 0,005% Tween 20) to typically 10 μg/ml before injection(1 mL) over the prepared surfaces. The immobilization resulted in protein levels ranging from 2000RU (response units, a measure of change in refractive index as result of plasmon resonance) to 5000 RU an expected range. The immobilization procedure was followed by injections of compound concentration series of VLX1570. Concentration series were set up with ×3 and ×2 dilution series ending with the highest concentration 200 μM for the consecutive measurements of changes of refractive index. Compounds were kept in stock concentrations of 10 mM in DMSO and diluted in running buffer down to the test concentration. R_max_ and K_D_ were calculated using Langmuir binding isotherms as provided in the Biacore software.

### Cell Viability Assay

Cell viability was monitored by the MTT (3-(4,5-dimethylthiazol-2-yl)-2,5-diphenyltetrazolium bromide) assay. For the MTT assay, cells were suspended at 5 × 10^5^ cells/ml, and 100 μl aliquots were dispended into 96-well microtiter plates and exposed to drugs as described using DMSO control. At the end of incubations, 10 μl of a stock solution of 5 mg/ml MTT (3-(4,5-dimethylthiazol-2-yl)-2,5-diphenyltetrazolium bromide), was added into each well, and the plates were incubated 4 hours at 37 °C. Formazan crystals were dissolved with 100 μl of 10% SDS/10 mM HCl solution overnight at 37 °C. Since MTT assays are affected by mitochondrial activity, and since OXPHOS is affected by VLX1570 (unpublished observation), we used the acid phosphatase method^49^ to determine cell viability in some experiments. After washing twice with PBS, cells were lysed in 100 μl of 0.1 M sodium acetate, 0,1% Triton X-100, p-nitrophenylphosphate (Pierce Biotechnology Inc, Rockford, IL) and incubated for 90 min at 37 °C. At the end of the incubation, 10 μl NaOH was added to each well and A_405_ was determined.

### siRNA Electroporation

Electroporation of OPM2 cells were performed as previously described^50^ with slight modification. In brief 4 × 10^6^ cells were suspended in RPMI media containing 6 μM siRNA against UCHL5 or USP14 or a scrambled control. Cells were electroporated at 180 V using an exponential pulse decay and 1000 μF capacitance and immediately transferred to complete media.

### Apoptosis Assays

Quantification of apoptosis by Annexin-V and propidium iodide staining and fluorescence-activated cell sorting (FACS) analysis was performed. Cells were cultured at density of 5 × 10^5^ cells/ml, then exposed to drugs as described using DMSO as control for 6 or 18 h. Cells were harvested and washed in 1× PBS twice, without fixing, which followed to stain with fluorescein-conjugated annexin-V and PI (BD-Biosciences). The accumulation of caspase-cleaved keratin-18 (K18- Asp396) was determined by the M30-Apoptosense^®^ ELISA^51^ as recommended by the manufacturer (PEVIVA-VLVbio, Stockholm, Sweden).

### Cell proliferation assay

Cell proliferation was measured using the Cell trace^TM^ CSFE cell proliferation kit. Cells were suspended at 5 × 10^5^ cells/ml, incubated with CellTrace^TM^ reagent for 20 minutes and exposed to the drug at indicated concentrations using DMSO as a control. After 72 h cells were collected, washed twice in 1× PBS, fixed with 3,7% formaldehyde and analysed by flow cytometry.

### Immunohistochemical staining

Tissue sections were deparaffinized with xylene, rehydrated and microwaved and then incubated with primary antibodies diluted in 1% (wt/vol) BSA and visualized by standard avidin–biotin–peroxidase complex technique (Vector Laboratories, Burlingame, CA, USA). Counterstaining was performed with Mayer’s haematoxylin. Slides were mounted in Vectashield containing DAPI and images recorded in a Zeiss Axioplan 2 fluorescence microscope. Antibodies: KI67 Dako #M7240 (1:100)(using Rodent Block (Biocare-RBM961) and Envision Plus anti-mouse labeled polymer (Dako K4001); Ub-K48 (Millipore-05-1307) (1:100) (serum free Protein block (Dako-X0909)) and Envision + anti-rabbit labeled polymer (Dako-K4003); CXCR4 (Sigma-C3116) (1:400) (Dako-X0909) and Envision + anti-rabbit labeled polymer (Dako-K4003); cleaved casapse-3 (Cell Signaling-9664) (1:100) (Dako-X0909) and Envision + anti-rabbit labeled polymer (Dako-K4003).

### Multiple myeloma animal models

Two different multiple myeloma models were used. Experimental protocols and methods were performed by Accelera (Nerviano, Italy) in accordance with guidelines and with permission from the local ethics committee (Nerviano Medical Sciences (NMS) Ethic Committee). All experimental protocols were approved by the Nerviano Medical Sciences (NMS) Ethic Committee. Study director was Marina Ciomei. SCID female mice were obtained from Charles River, Italy. Body weights at the day of tumor implant were between 19 and 22 grams. Treatment started at day 7 after injection of tumor cells and continued for 10 consequtive days. Mice were monitored daily for mortality and clinical signs. In the KMS-11 model, antitumor efficacy was evaluated in terms of survival increase respect to control. In the RPMI8226 model, the growth of subcutaneous tumors was recorded. For imaging, mice were injected intraperitoneally with 150 mg/kg d-luciferin (Promega) followed by anesthetization in 2–3% isoflurane atmosphere. After 10–12 minutes of biodistribution time, mice were imaged using a charge coupled device (CCD camera, Xenogen IVIS Lumina System) to evaluate the bioluminescence in the animal. Nose-cone isoflurane delivery system and heated stage for maintaining body temperature were used. A gray-scale image of the mice was captured, followed by an overlay of a bioluminescence map representing the spatial distribution of photons detected from cleaved luciferin in the cancer cells expressing luciferase. Signal intensity was quantified using a customized version of the IGOR Pro version 4.09A Software (WaveMetrics, Inc., Lake Oswego, OR) called Living Image version 3.00 (Xenogen). Photon emission was measured as whole-body radiance and the individual regions of interest (ROIs) were manually selected. Data were expressed as photon/second/cm^2^/steradian.

## Additional Information

**How to cite this article**: Wang, X. *et al.* The proteasome deubiquitinase inhibitor VLX1570 shows selectivity for ubiquitin-specific protease-14 and induces apoptosis of multiple myeloma cells. *Sci. Rep.* **6**, 26979; doi: 10.1038/srep26979 (2016).

## Supplementary Material

Supplementary Information

## Figures and Tables

**Table 1:** SPR and dissociation constants of drug binding to proteasomal DUBs and 26S proteasomes.

	USP14^(P)^ K_D_	UCHL5^(P))^ K_D_	USP14^(U)^ K_D_	USP14^(K)^ K_D_	26S^(V)^ K_D_	26S^(V)^ + USP14^(K)^ K_D_
NTA chip						
VLX1570	8.1 μM	14 μM	1.5 μM	18 μM	–	–
CM7 chip						
VLX1570	–	–	–	35 μM	25 μM	35 μM

Source of proteins in parenthesis: ProSpec (P), Ubiquigent (U), Karolinska Institute Protein Science Facility (K) and VIVA Bioscience (V).

**Table 2:** VLX1570 binding to immobilised 26S proteasome (VIVA Biosciences) analysed by surface plasmon resonance (SPR) using Biacore T200 and CM7 chip.

Immobilised protein on chip	Average protein load (RU)	VLX1570 binding Relative RU × 100 +/− S.D.
26S proteasome	8500	0.21 ± 0.09
26S proteasome Ub-VS	10850	0.04 ± 0.01

Protein load on chip and relative light units(RU)×100 ± SD (defined as average of the ratio of VLX1570 RU divided by individual protein load RU) are listed. Values are the average of two chip experiments repeated three times.

**Table 3:** Antiproliferative activities of VLX1570, b-AP15 and bortezomib on multiple myeloma cells.

Compound	VLX1570	b-AP15	bortezomib
KMS-11	43 ± 2	83 ± 3	67 ± 3
RPMI8226	74 ± 2	150 ± 14	84 ± 5
OPM-2	126 ± 3	160 ± 3	32 ± 1
OPM-2-BZ^R^	191 ± 1	268 ± 12	204 ± 10

Shown in this table are IC_50_ values in nanomolar (nM) determined over 72 hours using the MTT assay.
