# Corrigendum: The proteasome deubiquitinase inhibitor VLX1570 shows selectivity for ubiquitin-specific protease-14 and induces apoptosis of multiple myeloma cells

**DOI:** 10.1038/srep30667

**Published:** 2016-07-29

**Authors:** Xin Wang, Magdalena Mazurkiewicz, Ellin-Kristina Hillert, Maria Hägg Olofsson, Stefan Pierrou, Per Hillertz, Joachim Gullbo, Karthik Selvaraju, Aneel Paulus, Sharoon Akhtar, Felicitas Bossler, Asher Chanan Khan, Stig Linder, Padraig D’Arcy

Scientific Reports 6: Article number: 2697910.1038/srep26979; published online: 06 06 2016; updated: 07 29 2016

The Acknowledgements section in this Article is incomplete:

“We are grateful to Hans Rosén and Per Hagmar (Vivolux AB) for discussions and study support and to Marina Ciomei (Accelera, Nerviano, Italy) for performance of animal studies. We are grateful to Ingrid Holmberg for artwork. We acknowledge Cancerfonden, Radiumhemmets forskningsfonder, Vetenskapsrådet, Barncancerfonden for support.”

should read:

“We are grateful to Hans Rosén and Per Hagmar (Vivolux AB) for discussions and study support and to Marina Ciomei (Accelera, Nerviano, Italy) for performance of animal studies. We are grateful to Ingrid Holmberg for artwork. We acknowledge Cancerfonden, Radiumhemmets forskningsfonder, Vetenskapsrådet, Barncancerfonden for support. We would like to acknowledge support from the Mayo Clinic Multiple Myeloma SPORE (P50 CA186781-01A1, Aneel Paulus) and the University of Iowa/Mayo Clinic Lymphoma SPORE (P50 CA097274, Aneel Paulus)”.

