# Supplementary material for: The proteasome deubiquitinase inhibitor VLX1570 shows selectivity for ubiquitin-specific protease-14 and induces apoptosis of multiple myeloma cells
Source: Sci Rep. 2016 Jun 6;6:26979. doi: 10.1038/srep26979 (PMC4893612; doi:10.1038/srep26979)
Supplement: Supplementary Information [file srep26979-s1.pdf]

Supplementary material

The proteasome deubiquitinase inhibitor VLX1570 shows selectivity for ubiquitin-specific protease-14 and induces apoptosis of multiple myeloma cells

Xin Wang, Magdalena Mazurkiewicz, Ellin-Kristina Hillert,
Maria Hägg Olofsson, Stefan Pierrou, Per Hillertz, Joachim Gullbo,
Karthik Selvaraju, Aneel Paulus, Sharoon Akhtar,
Felicitas Bossler, Asher Chanan Khan, Stig Linder and Padraig D'Arcy

Suppl Fig. 1a

b-AP15

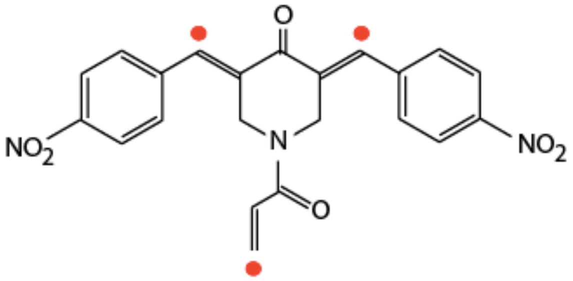

VLX1570

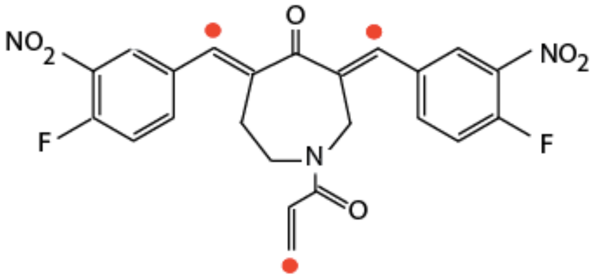

RA-9

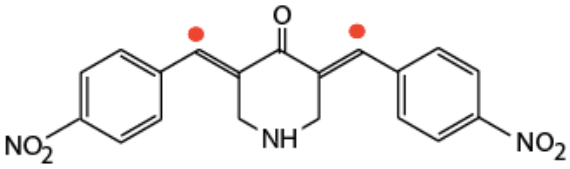

b-AP113

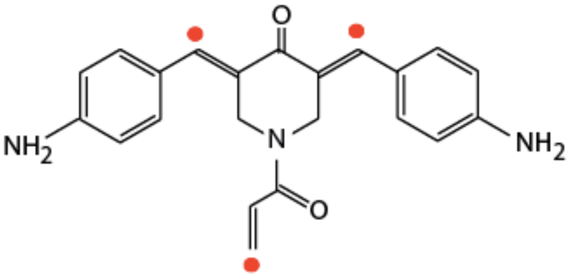

IU1

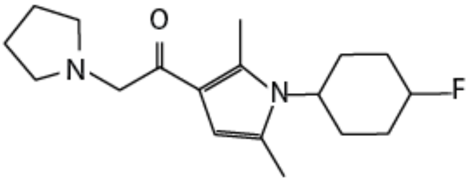

WP1130

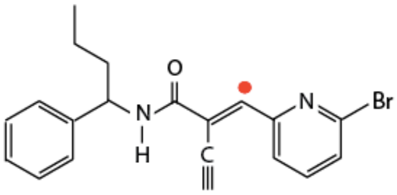

RA190

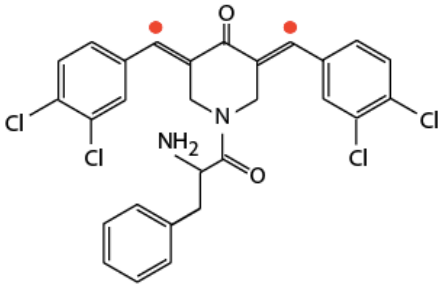

Structures of compounds mentioned in this article. Potential Michael acceptors (α, β–carbonyls) are indicated with filled red circles.

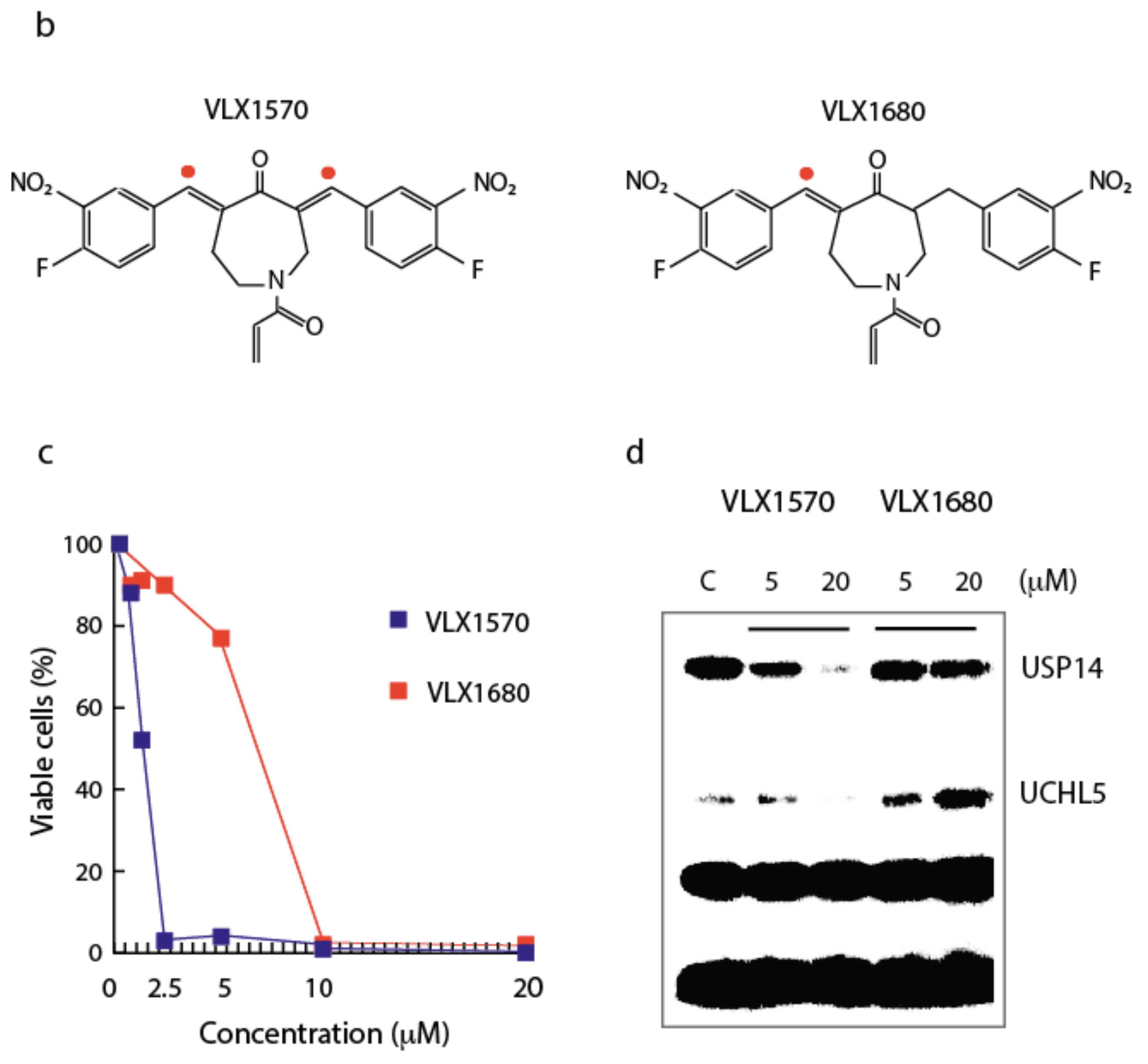

(b) Both α, β–carbonyls (red circles) in the long conjugated system are required for biological activity. VLX1680 contains only one potential Michael acceptor. Both the cytotoxic activity (c) and the DUB inhibitory activity (d) of VLX1680 is lower than that of VLX1570.
DUB enzyme activity was determined using Ub-VS labeling and survival was determined by MTT assay.

Suppl Fig. 3

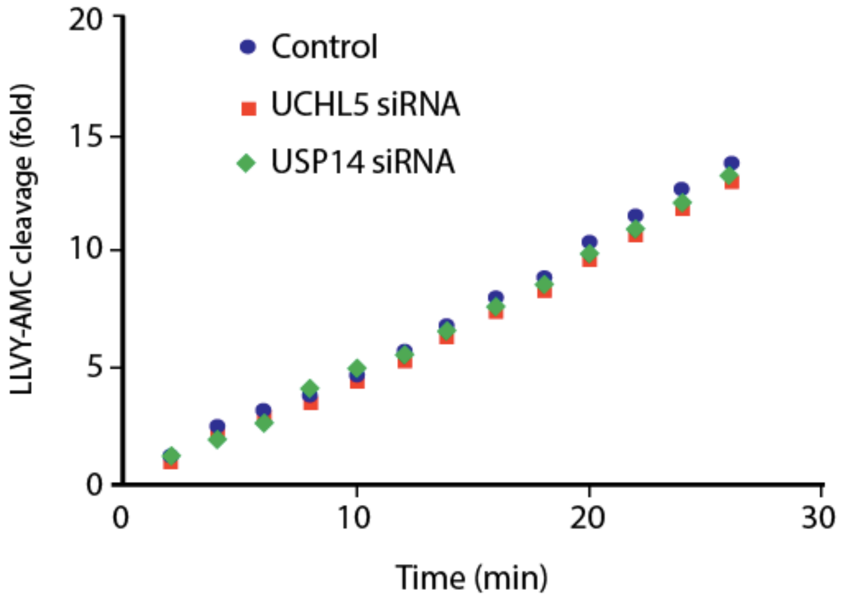

Knock-down of USP14 or UCHL5 does not inhibit 20S proteasome activity. OPM-2 cells were transfected with siRNA to USP14 or UCHL5 or with scrambled siRNA are cell extracts were prepared at 48 hours. LLVY-AMC cleavage activity was then assayed in cell extracts.

Suppl Fig. 4a

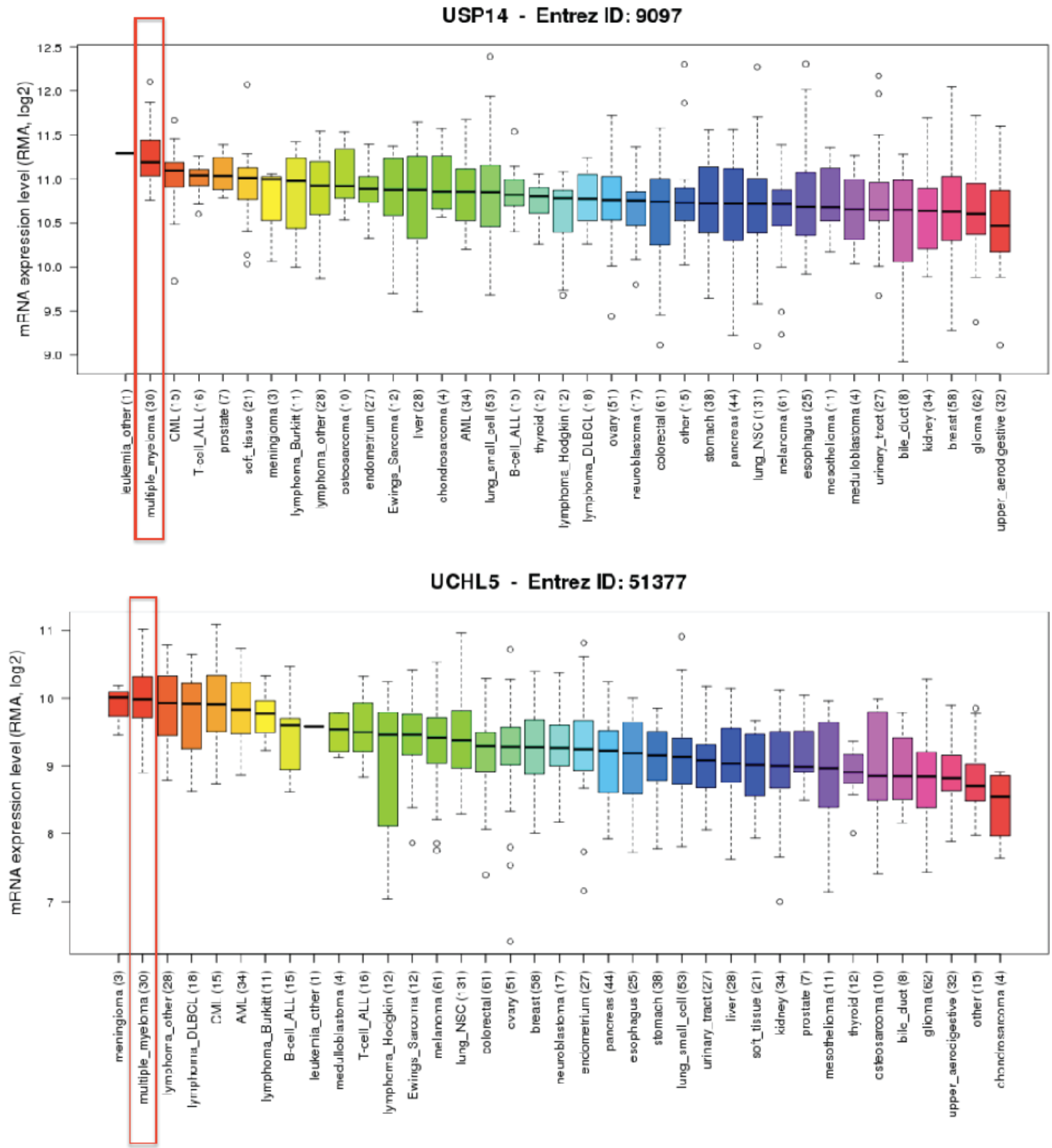

USP14 and UCHL5 expression in cancer cell line panels (from the Broad-Novartis Cancer Cell Line Encyclopedia (https://www.broadinstitute.org/ccle/home)).

Suppl Fig. 4

b

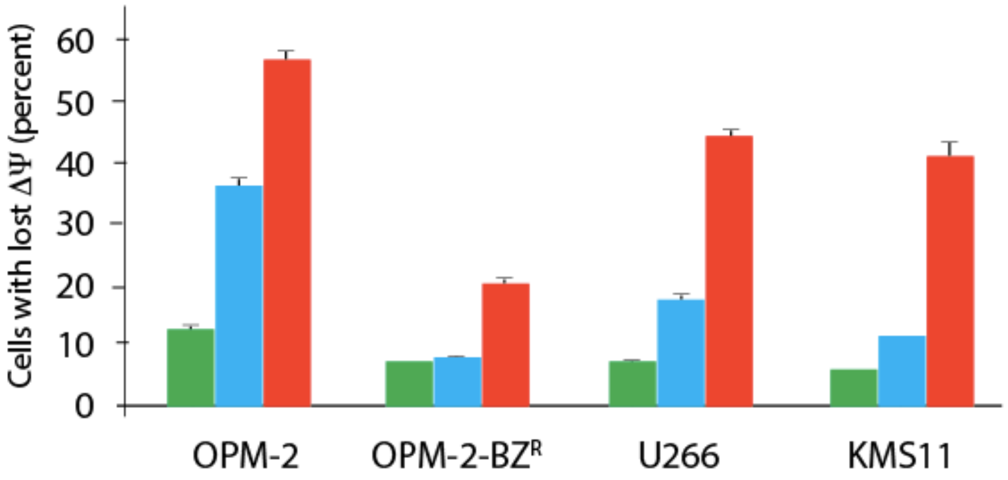

c

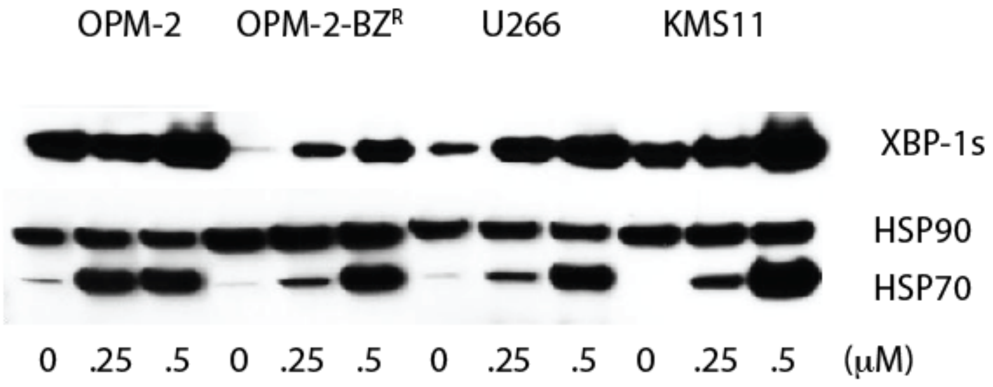

(b): Induction of ER stress by VLX1570. The polypeptide product of the spliced form of XBP-1 is induced by VLX1570 in three different MM cell lines (OPM-2, KMS11 and U266). An equal amount of protein was loaded onto each slot.

(c): VLX1570 elicits mitochondrial membrane depolarization in MM cell lines. Mitochondrial membrane depolarization was determined by staining with TMRE and quantified by flow cytometry as described (Chitta et al., Br J Haematology 169,377-90).

Supplementary Fig. 4

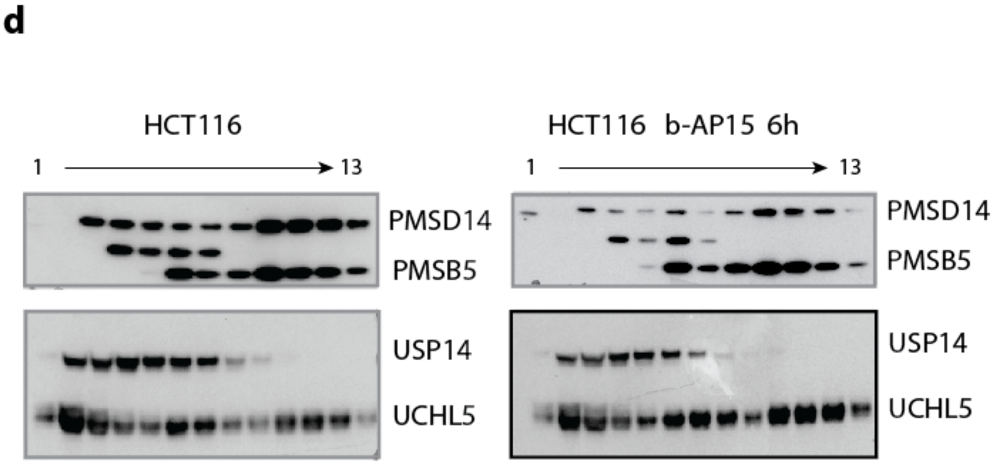

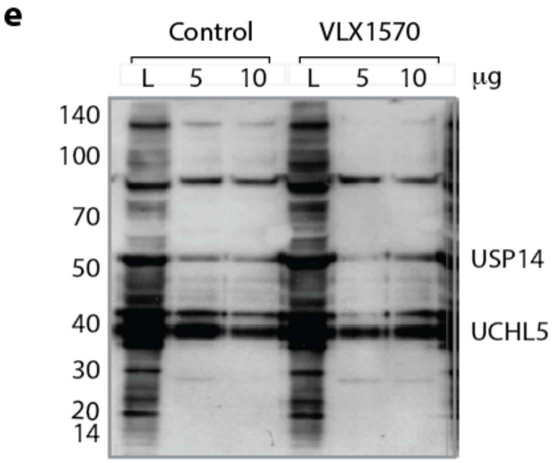

(d) HCT116 cells were exposed to 1 μM VLX1500 for 6h and cell lysates were subjected to glycerol gradient centrifugation. Samples from each fraction were subjected to immunoblotting using the indicated antibodies. Note the similar sedimentation profiles of PSMD14 (19S) and PSMB5 (20S) in cells exposed to b-AP15 and control cells and note the similar sedimentation profiles of USP14 and UCHL5.

(e) Purification of proteasomes from HEK293 cells expressing His-tagged Rpn11. Cells were exposed to VLX1570 where indicated. Immunoblots were probed with antibodies to USP14 and UCHL5. The amount of protein loaded is indicated.

Suppl. Fig. 5

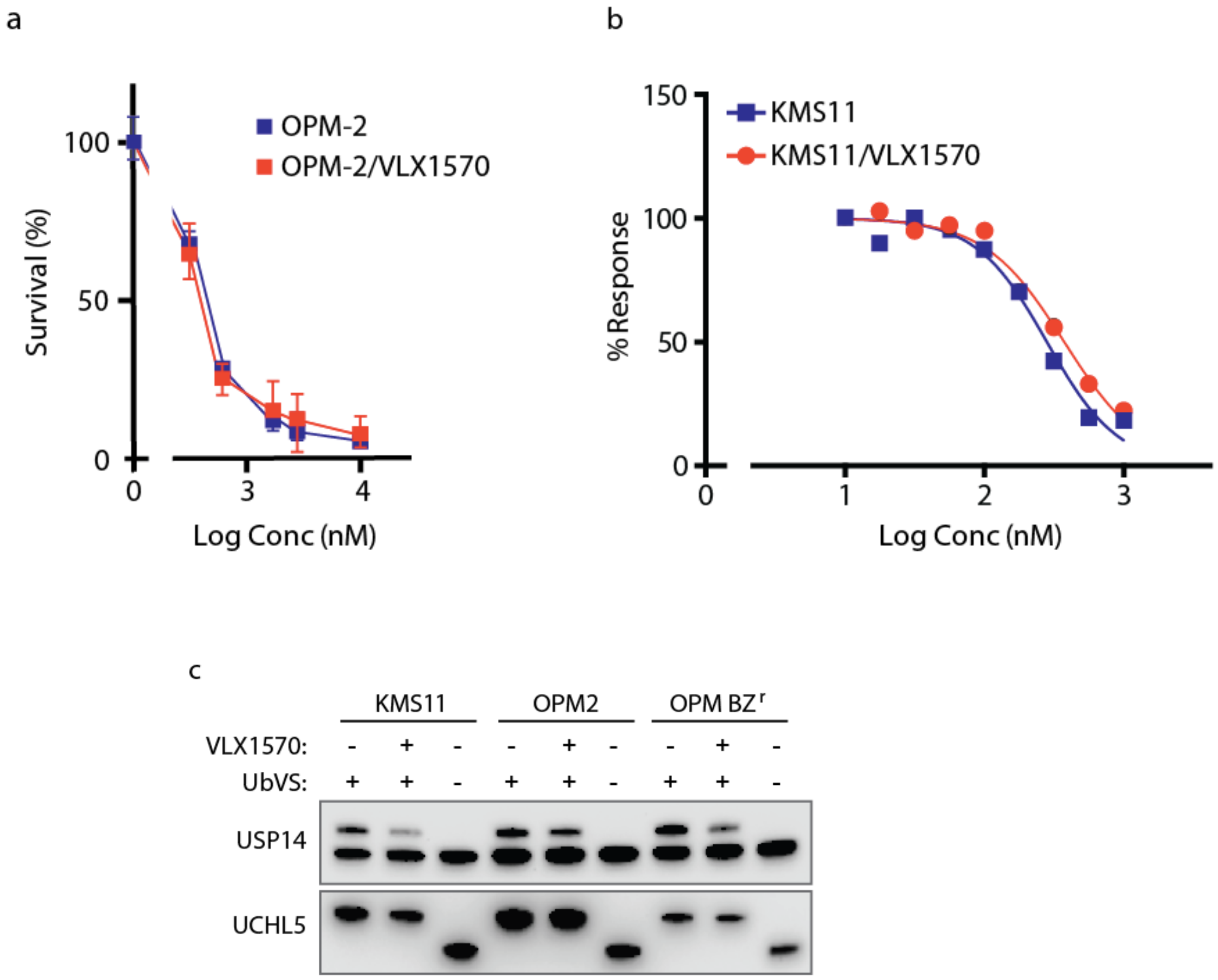

(a, b) OPM-2 or KMS-11 cells were exposed to increasing concentrations of VLX1500 in order to select for resistant cells. Despite several months of incubation of the presence of drug, selected cells did not display detectable resisistance.

(c) Proteasome DUB activity of KMS11, OPM-2 and OPM-BZ^r cells. Note the similar levels of DUB activity in KMS11 and OPM-2 cells and note that UPS14 activity in OPM-BZ^r cells was sensitive to VLX1570.

Suppl Fig. 5d

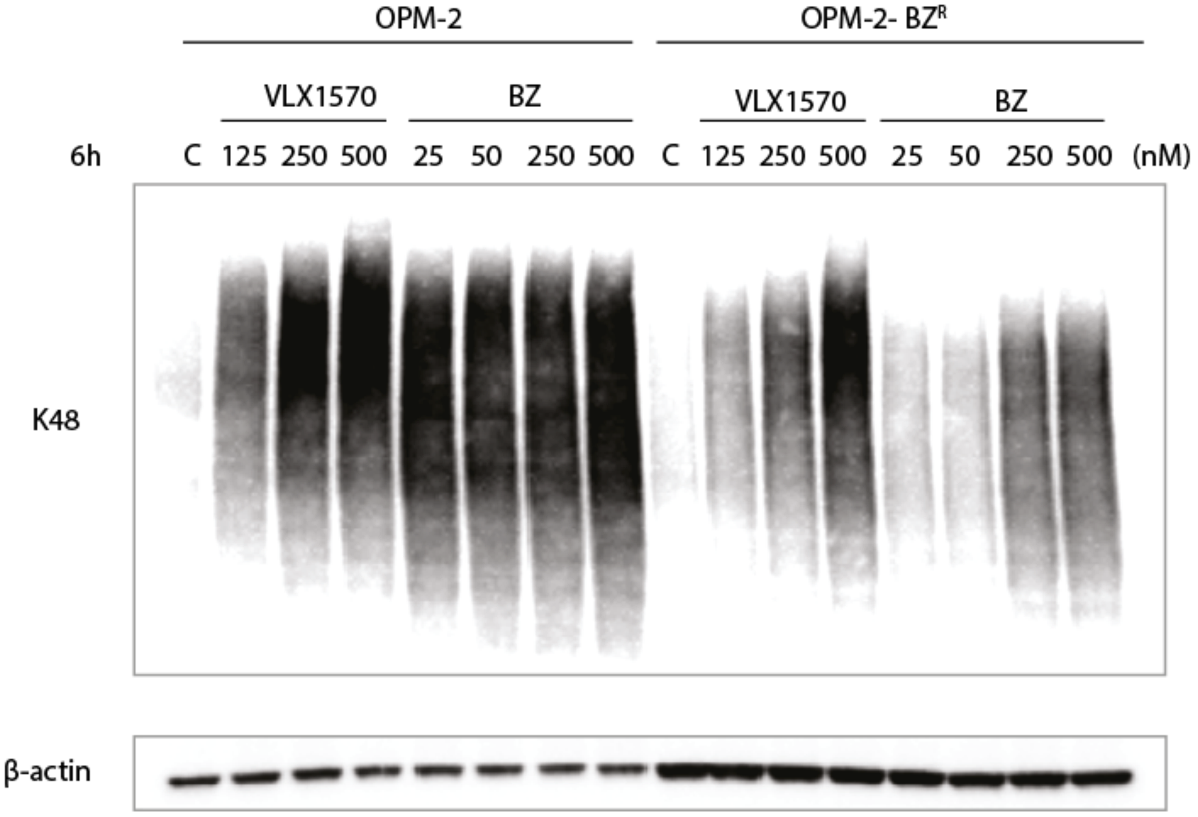

OPM-2 and OPM-2-BZ^R cells were exposed to VLX1570 or bortezomib for 6 hours and processed for immunoblotting. Note that when samples are blotted to the same filter weaker signals for Ub-K48 are observed in OPM-2-BZ^R cells exposed to VLX1570 and bortezomib.

Suppl Fig. 5e

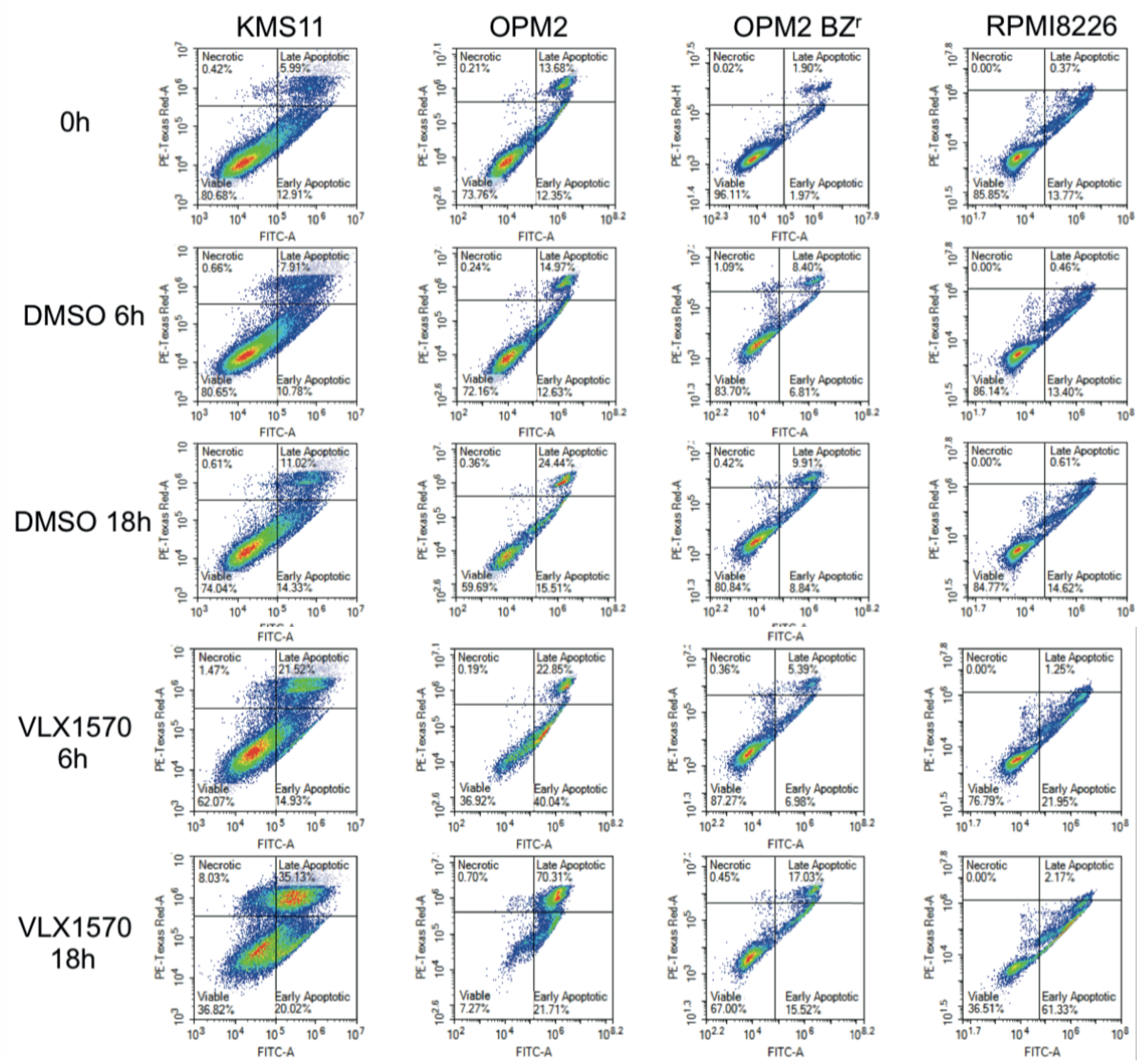
KMS11
OPM2
OPM2 BZr
RPMI8226
0h
DMSO 6h
DMSO 18h
VLX1570 6h
VLX1570 18h
PE-Texas Red-A
PE-Texas Red-H
FITC-A
Necrotic 0.42%
Late Apoptotic 5.99%
Viable 80.68%
Early Apoptotic 12.91%
Necrotic 0.21%
Late Apoptotic 13.68%
Viable 73.76%
Early Apoptotic 12.35%
Necrotic 0.02%
Late Apoptotic 1.90%
Viable 96.11%
Early Apoptotic 1.97%
Necrotic 0.00%
Late Apoptotic 0.37%
Viable 85.85%
Early Apoptotic 13.77%
Necrotic 0.66%
Late Apoptotic 7.91%
Viable 80.65%
Early Apoptotic 10.78%
Necrotic 0.24%
Late Apoptotic 14.97%
Viable 72.16%
Early Apoptotic 12.63%
Necrotic 1.09%
Late Apoptotic 8.40%
Viable 83.70%
Early Apoptotic 6.81%
Necrotic 0.00%
Late Apoptotic 0.46%
Viable 86.14%
Early Apoptotic 13.40%
Necrotic 0.61%
Late Apoptotic 11.02%
Viable 74.04%
Early Apoptotic 14.33%
Necrotic 0.36%
Late Apoptotic 24.44%
Viable 59.69%
Early Apoptotic 15.51%
Necrotic 0.42%
Late Apoptotic 9.91%
Viable 80.84%
Early Apoptotic 8.84%
Necrotic 0.00%
Late Apoptotic 0.61%
Viable 84.77%
Early Apoptotic 14.62%
Necrotic 1.47%
Late Apoptotic 21.52%
Viable 62.07%
Early Apoptotic 14.93%
Necrotic 0.19%
Late Apoptotic 22.85%
Viable 36.92%
Early Apoptotic 40.04%
Necrotic 0.36%
Late Apoptotic 5.39%
Viable 87.27%
Early Apoptotic 6.98%
Necrotic 0.00%
Late Apoptotic 1.25%
Viable 76.79%
Early Apoptotic 21.95%
Necrotic 8.03%
Late Apoptotic 35.13%
Viable 36.82%
Early Apoptotic 20.02%
Necrotic 0.70%
Late Apoptotic 70.31%
Viable 7.27%
Early Apoptotic 21.71%
Necrotic 0.45%
Late Apoptotic 17.03%
Viable 67.00%
Early Apoptotic 15.52%
Necrotic 0.00%
Late Apoptotic 2.17%
Viable 36.51%
Early Apoptotic 61.33%

Suppl Fig. 5f

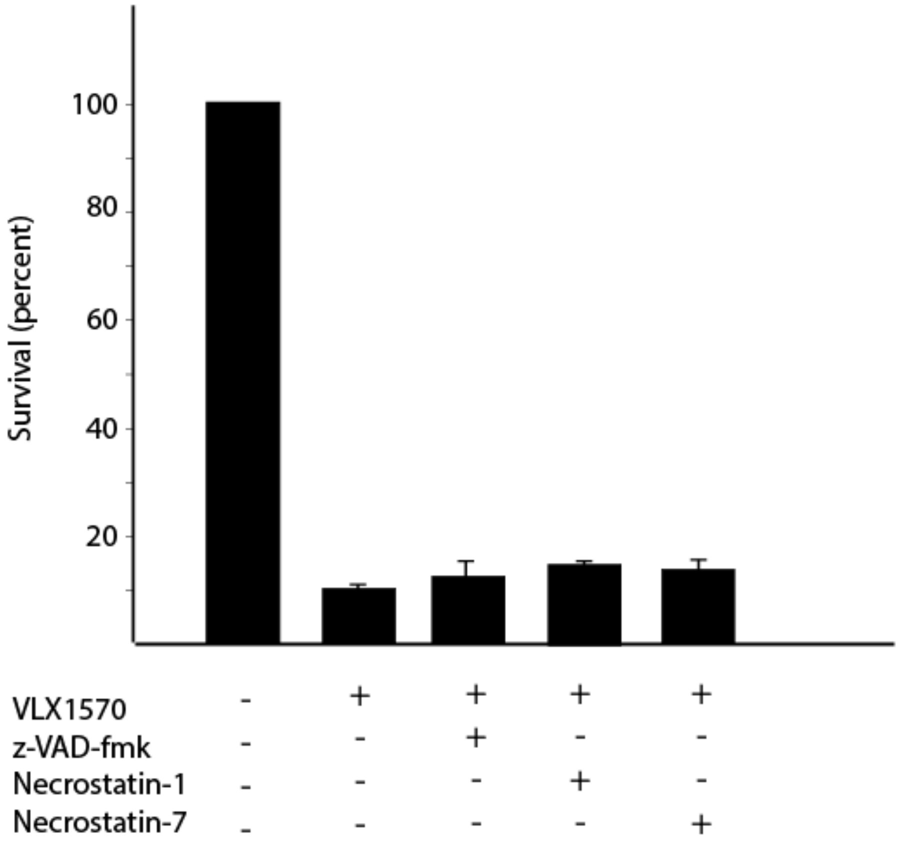

Calculation of survival of HCT116 cells exposed to VLX1570 in the presence of absence of caspase or necroptosis inhibitors. VLX1570 treatment was for 24 hours; z-VAD-fmk was used at 20 μM and necrostatin-1 and -7 at 20 μM. Survival was determined using an IncuCyte instrument (Essen Bioscience).

Suppl. Fig. 7a

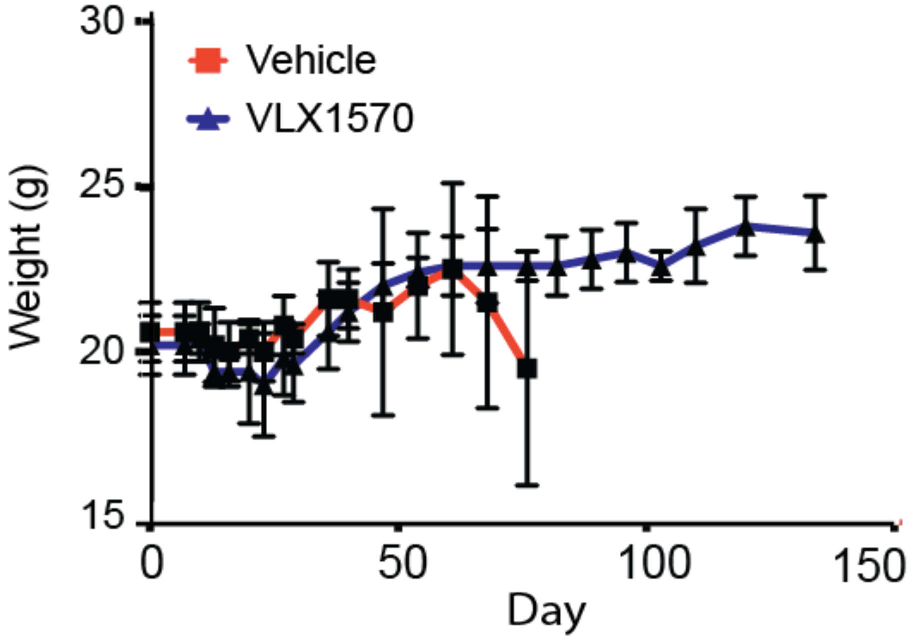

Weight of animals injected with KMS-11 multiple myeloma cells. Note the increase in the mean body weights in the VLX1570-treated group and the decrease in the control group (related to disease progression)

Suppl Fig. 7b

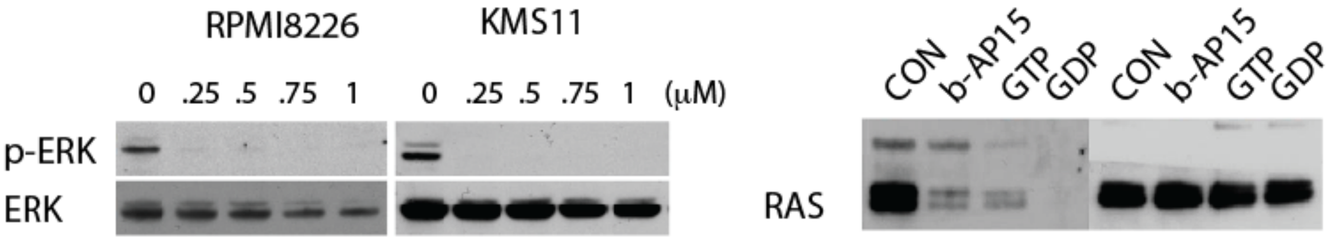

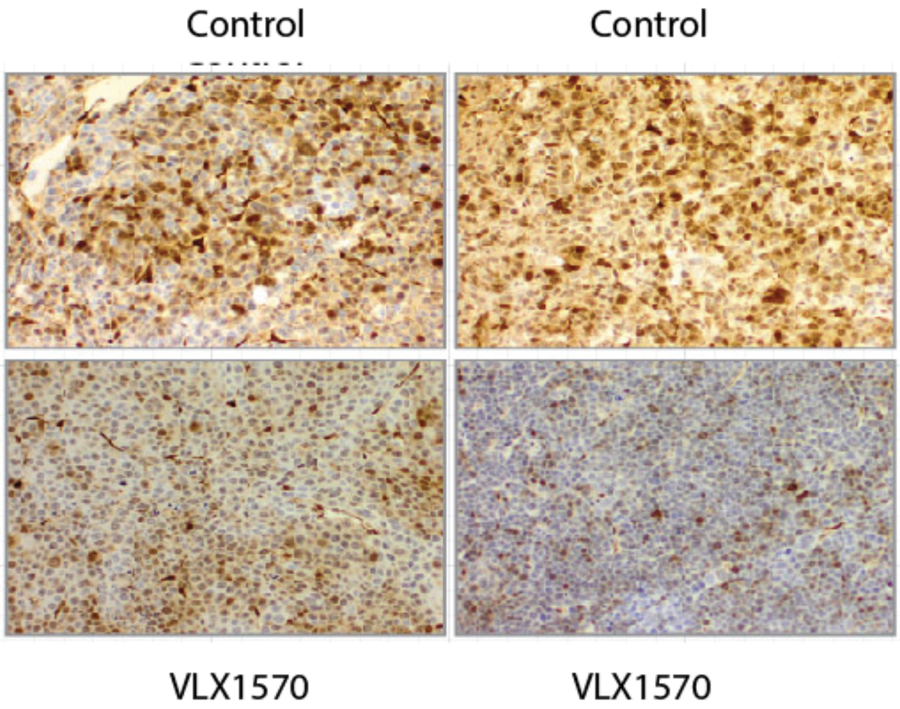

(upper panel; left) Decrease in ERK phosphorylation in multiple myeloma cells exposed to VLX1500.

(upper panel; right) Ras activation assay of KMS11 cells; control (Con) or treated for 3 hours with 1 μM VLX1500 (b-AP15). Active GTP-bound Ras was pulled down. Untreated cell lysate was loaded with GTPγS (GTP) and GDP as control for the pull-down of GTP-Ras. Immunoblot for Ras of the pull-down (Ras-GTP) and the total cell lysate (Input) is shown. Ras activity was determined using a Ras activation assay kit (Upstate) according to the manufacturers' instructions.

(lower panel) ERK phosphorylation in vivo in sections from RMI8226 tumors following treatment with VLX1570. Mice were treated with 3 mg/kg VLX1570 24 hours prior to sacrifize. Tumors were fixed, sectioned and stained with an antibody to phosphorylated ERK.

Suppl Fig. 8

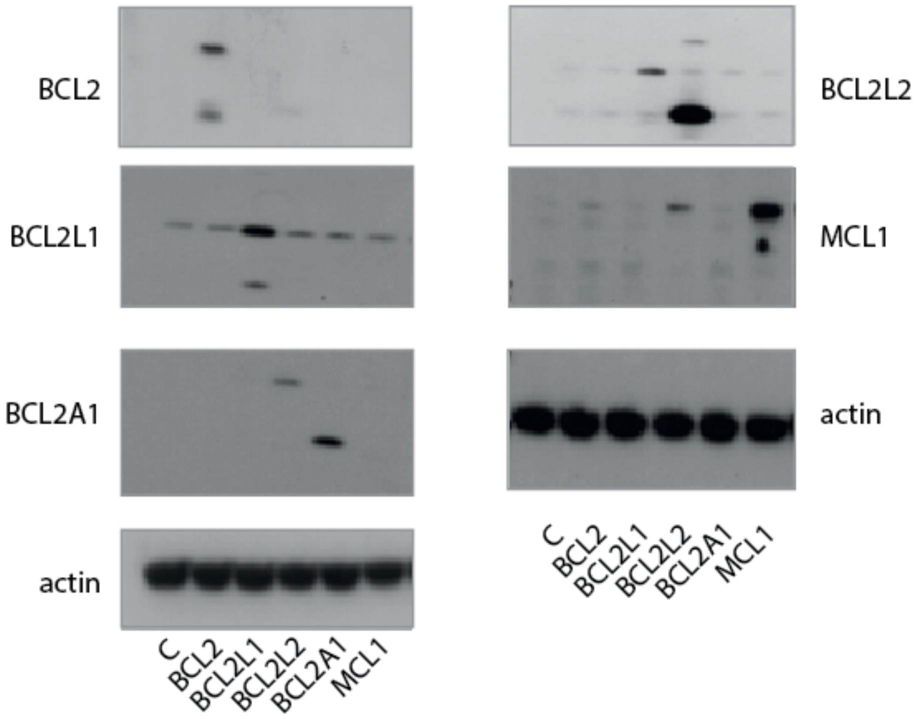

Uncropped images for Fig. 5d. The same extracts were electrophoresed on two separated PAGE gels and immunoblotted. Fig 5d was assembled from these films. Note the strong expression of BCL2L2. The corresponding cells showed very inconsistent apoptosis induction between different experiments when exposed to drugs (BCL2L2 did not, however, increase cell viability). We chose not to present data for BCL2L2.
